# LFA-1 activates focal adhesion kinases FAK1/PYK2 to generate LAT-GRB2-SKAP1 complexes that terminate T-cell conjugate formation

**DOI:** 10.1038/ncomms16001

**Published:** 2017-07-12

**Authors:** Monika Raab, Yuning Lu, Karsten Kohler, Xin Smith, Klaus Strebhardt, Christopher E. Rudd

**Affiliations:** 1 Cell Signaling Section, Department of Pathology, Tennis Court Road, University of Cambridge, Cambridge CB2 1QP, UK; 2 Department of Obstetrics and Gynaecology, School of Medicine, J.W. Goethe-University, Theodor-Stern-Kai 7, 60590 Frankfurt, Germany; 3 Division of Immunology-Oncology, Research Center, Maisonneuve-Rosemont Hospital, Montreal, Quebec, Canada H1T 2M4; 4 Département de Medicine, Université de Montréal, Montreal, Quebec, Canada H3C 3J7

## Abstract

Lymphocyte function-associated antigen 1 (LFA-1) affinity and avidity changes have been assumed to mediate adhesion to intercellular adhesion molecule-1 for T-cell conjugation to dendritic cells (DC). Although the T-cell receptor (TCR) and LFA-1 can generate intracellular signals, the immune cell adaptor protein linker for the activation of T cells (LAT) couples the TCR to downstream events. Here, we show that LFA-1 can mediate both adhesion and de-adhesion, dependent on receptor clustering. Although increased affinity mediates adhesion, LFA-1 cross-linking induced the association and activation of the protein-tyrosine kinases FAK1/PYK1 that phosphorylated LAT selectively on a single Y-171 site for the binding to adaptor complex GRB-2-SKAP1. LAT-GRB2-SKAP1 complexes were distinct from canonical LAT-GADs-SLP-76 complexes. LFA-1 cross-linking increased the presence of LAT-GRB2-SKAP1 complexes relative to LAT-GADs-SLP-76 complexes. LFA-1-FAK1 decreased T-cell-dendritic cell (DC) dwell times dependent on LAT-Y171, leading to reduced DO11.10 T cell binding to DCs and proliferation to OVA peptide. Overall, our findings outline a new model for LFA-1 in which the integrin can mediate both adhesion and de-adhesion events dependent on receptor cross-linking.

## Abstract (web summary)

The T-cell integrin LFA-1 binds ICAM-1 on antigen presenting cells to affect TCR-MHC interactions. Here the authors show detailed mechanics of how LFA-1 ligation affects T-cell conjugation to dendritic cells to regulate adhesion and de-adhesion of these cells in the context of antigen presentation.

T-cell antigen receptor (TCR) engagement activates a protein tyrosine activation cascade that is accompanied by the formation of multi-protein signalling complexes for T-cell activation^1^,^2^,^3^. These cascades are initiated by p56^lck^, ZAP-70 and Tec-family protein tyrosine kinases (PTKs) and various effector molecules^1^,^2^,^3^,^4^,^5^,^6^,^7^. Adaptors are proteins with sites and modules that mediate the formation of complexes that integrate signals in cells. Of these adaptors, the linker for the activation of T cells (LAT) and SLP-76 are phosphorylated by ZAP-70 (refs 8, 9). LAT-deficient mice are arrested in thymocyte development^10^, whereas in deficient Jurkat cells, LAT is needed for calcium mobilization and the optimal activation of downstream extracellular regulated kinases (ERKs) and expression of CD69 (refs 10, 11, 12). ZAP-70 phosphorylates multiple sites (Y-132, Y-191, Y-171 and Y-226) on LAT, which in turn recruit phospholipase Cγ1 (PLCγ1) and adaptors growth-factor-receptor-bound protein 2 (GRB2) and GRB2-related adaptor downstream of Shc (GADS)- SH2 domain containing leukocyte protein of 76kDa (SLP-76) (or lymphocyte cytosolic protein 2 (lcp2)^2^. LAT residue Y-132 binds to phospholipase C-γ1 (PLC-γ1), whereas residues Y-171 and Y-191 bind to GADs and GRB2 (refs 13, 14, 15). SLP-76 is recruited to LAT indirectly via its interaction with GADs^16^. GRB2 contains an SH2 domain flanked by amino-terminal and carboxy-terminal SH3 domains, and is involved in activation of the Ras and MAP kinase pathways. The GADs SH2 domain binds to phosphorylated LAT residues, whereas the SH3 domain binds to a non-canonical motif on SLP-76 (refs 16, 17). SLP-76 binds in turn to adhesion-and degranulation-promoting adapter protein (ADAP) (HUGO designation: *Fyb*) and hematopoietic progenitor kinase 1 (refs 18, 19, 20, 21).

The integrin family is comprised of 18α and 8β subunits that form 24 hetero-dimeric integrins with different ligand specificities^22^. Lymphocyte function-associated antigen 1 (LFA-1) is expressed on T cells and binds to intercellular adhesion molecules (ICAM)-1 and -2 on major histocompatibility complex (MHC) bearing antigen-presenting cells (APCs)^23^. The integrin controls conjugate formation between T cells and antigen-presenting cells as well as the migration of T cells to sites of inflammation and within lymph nodes (LNs)^24^. LFA-1 can be activated by TCR and chemokine receptor ligation^25^. Increased affinity and clustering (that is, avidity) are thought to contribute to adhesion. Increased affinity is accomplished by the extension of the extracellular domain^26^. Disruption of conformational changes in the extracellular domain blocks adhesion^27^. Clustering has been thought to increase contact numbers and stabilize adhesion, although the full purpose of this event has yet to be proven. Previous studies have underscored the primacy of affinity over clustering in regulation of adhesion^27^.

Increased affinity is accomplished by the extension of the extracellular domain and separation of LFA-1 αL and β2 cytoplasmic subunits^28^. FERM-domain carrying talin and kindlins to the β-chain alters the conformation of the extracellular domains^29^. Adaptor protein RIAM (Rap1–GTP-interacting adapter molecule) facilitates talin localization^30^ and interacts with the cytoskeleton via profiling and Ena/VASP proteins. GTP-binding protein Rap1 and associated RapL bind to the α-chain, whereas RapL-deficient lymphocytes exhibit impaired adhesion and migration^31^.

In T cells, the immune cell adaptor SKAP1 (src kinase-associated phosphoprotein 1) (SKAP-55, src kinase-associated phosphoprotein-55) couples the T-cell receptor (TCR) to the activation of LFA-1 (refs 32, 33, 34, 35, 36). This is mediated in part by its interaction with RapL^37^,^38^. The Rap1-RapL complex fails to form in Skap1-deficient T cells, which correlates with reduced binding to ICAM-1 and conjugation with dendritic cells (DCs)^32^,^33^,^34^,^39^. SKAP1 is needed for RapL plasma membrane localization^37^,^38^. SKAP1 influences RIAM-talin localization at the T-cell interface with DCs, whereas a cleavage-resistant talin (L432G) restores conjugation^40^. SKAP-1 also binds to another immune cell adaptor termed ADAP^18^,^20^,^33^,^35^,^36^, whereas LFA-1 co-signals engage ADAP in the induction of morphology and motility changes^19^,^41^. Considerable evidence indicates that LFA-1 ligation can induce co-signals that influence TCR signalling^19^,^34^,^41^,^42^. LFA-1 activates the Jun activated kinases and p44/42MAPK pathways and prolongs TCR-mediated inositol phospholipid hydrolysis^43^.

Members of the focal adhesion kinase family also regulate cell adhesion and motility^44^. The family includes FAK1 (Focal Adhesion Kinase 1) and PYK2 (proline-rich tyrosine kinase-2). FAKs are comprised of an *N*-terminal FERM (band 4.1, ezrin, radixin, moesin homology) domain, a 40 residue linker region, a kinase domain, a 200 residue proline-rich region, and a *C*-terminal focal adhesion targeting domain^45^. The FERM and kinase domains form an auto-inhibitory interaction^45^, which is released in focal adhesions^46^. Focal adhesion kinases regulate focal adhesion contacts, motility and cell survival^47^. Non-lymphoid cells from FAK-deficient mice show enhanced focal adhesion contact formation and reduced cell motility^48^. FAK auto-phosphorylation at Tyr-397 is needed for kinase activation and acts to bind to the SH2-domain of p60^Src^ kinase^49^. TCR engagement induces FAK and PYK2 phosphorylation and translocation to the immunological synapse (IS)^50^,^51^,^52^.

The observation that increased LFA-1 affinity for adhesion precedes the clustering of the integrin caused us to question whether the cross-linking of LFA-1 operated solely to increase LFA-1 adhesion, or whether it could also auto-regulate to terminate adhesion and the interaction between T cells and APCs. Given that LFA-1 generates co-signals, it is also of interest whether LFA-1 can interface with TCR signalling by altering the make-up of the LAT adaptor complex. Here, we show that while affinity changes increase LFA-1 mediated adhesion, the cross-linking of LFA-1 activated associated focal adhesion kinases FAK/PYK2 to phosphorylate LAT Y-171 for a reduction in the contact times of T cells with DCs. These findings support an ‘auto-regulatory on-off model’ for LFA-1 where the integrin can mediate both adhesion and de-adhesion, dependent on receptor cross-linking.

## Results

### LFA-1 induces SKAP1 binding to LAT

**Figure 1:**
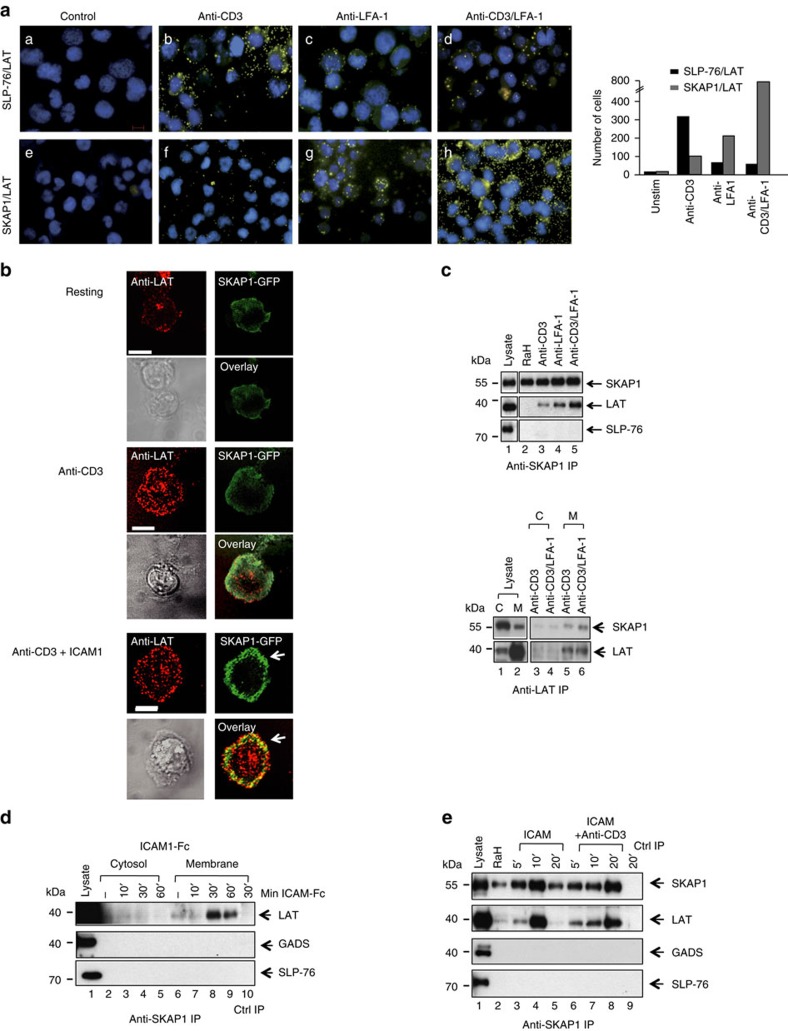
LFA-1 induced SKAP1-LAT and reduced LAT-SLP-76 complexes. (**a**) *In situ* proximity analysis shows anti-LFA-1 induced SKAP1-LAT proximity. Murine DC27.10T-cells were ligated with anti-CD3 and/or LFA-1 followed by *in situ* proximity analysis for SLP-76 and LAT (upper panels) or SKAP1 and LAT (lower panels) (*n*=4). (**b**) LFA-1 cross-linking increases SKAP1-LAT micro-cluster colocalization. Jurkat T cells transfected with mCherry-LAT and GFP-SKAP1 were stimulated on anti-CD3 or ICAM1/CD3 coated cover slips. Micro-cluster formation was monitored by confocal microscopy. Upper panel: resting; middle panel: anti-CD3; lower panel anti-CD3/ICAM1. Scale bar corresponds to 10 μM. (*n*=3) (**c**) SKAP1 co-precipitates LAT and vice versa in response to anti-LFA-1. DC27.10 T cells were ligated with anti-CD3 and/or anti-LFA-1 for 5 min followed by membrane preparation and precipitation with anti-SKAP1 and blotting with anti-SKAP1, anti-LAT and anti-SLP-76 (upper panels). Conversely, cells were separated into cytosol (lanes 3,4) and membranes (lanes 5,6) and precipitated with anti-LAT followed by blotting with anti-LAT or anti-SLP-76 (*n*=3). (**d**) ICAM1 binding to LFA-1 increases the binding of SKAP1 to LAT. DC27.10T-cells were ligated with ICAM1-Fc alone on plates (2 μg ml^−1^) for various times followed by anti-SKAP1 precipitation and blotting with either anti-LAT, anti-SLP-76 or anti-GADs (*n*=3) (**e**) Anti-LFA-1 induced LAT-SKAP1 in human peripheral primary T-cells. Human resting T-cells ligated with plate bound ICAM1-Fc (2 μg ml^−1^) or ICAM1-Fc plus soluble anti-CD3 (1 μg ml^−1^) for various times followed by anti-SKAP1 precipitation and blotting with anti-SKAP1, anti-LAT, anti-GADS or anti-SLP-76 (*n*=3).

The LAT binds to PLCγ1, GADs-SLP-76 and GRB2 for the activation of T cells^2^,^10^. To assess whether the LAT complex could be altered by LFA-1, T cells were ligated with either anti-CD3 or anti-LFA-1 in an *in situ* proximity ligation assay (PLA) (Fig. 1a). Unless otherwise stated, both anti-CD3 and anti-LFA-1 were bivalent and therefore cross-link their respective receptors. Antibodies to LAT, SLP-76 and SKAP1 were employed using isotype-specific antibodies with the DuolinkTM detection system^53^. Anti-CD3 induced SLP-76-LAT proximity signals as shown by an increase in fluorescent dots (Fig. 1a, panel b, also right histogram). Anti-LFA-1 induced no SLP-76-LAT proximity signals (Fig. 1a, panel c), whereas the combination of anti-CD3/LFA-1 reduced the signal compared with anti-CD3 alone (Fig. 1a, panel d). Interestingly, by contrast, anti-LFA-1 induced a moderate PLA signal between LAT and SKAP1 (Fig. 1a, panel g; see right histogram), whereas anti-LFA-1 and anti-CD3 produced the strongest PLA signal between SKAP-1 and LAT (Fig. 1a, panel h). Anti-CD3 alone induced a relatively weak proximity signal between LAT and SKAP1 (Fig. 1a, panel f). These results showed that LFA-1 cross-linking increased the proximity of LAT and SKAP1 either alone or in conjunction with anti-CD3.

We next assessed whether LFA-1 could promote SKAP1 co-clustering with LAT by immunofluorescent time-lapse confocal microscopy (Fig. 1b). Micro-clusters of LAT are induced in response to anti-CD3 ligation^54^,^55^. We previously also reported that vesicular LAT interacts with surface SLP-76 (ref. 56). Jurkat T cells transfected with LAT-cherry and SKAP1-GFP were imaged on anti-CD3 or anti-CD3-ICAM1-Fc-coated cover slips and monitored as previously described^56^,^57^,^58^. Anti-CD3 induced LAT-cherry clusters by 2 min ligation in the central and peripheral contact regions (Fig. 1b, left middle panels). Anti-CD3/ICAM-1 induced a similar distribution of LAT-cherry clusters, although usually at slightly lower levels (Fig. 1b, left lower panel). By contrast, Although anti-CD3 induced a diffuse array of SKAP1-GFP around the peripheral region of the cell (Fig. 1b, middle right panels), co-ligation of LFA-1 by ICAM1 induced markedly larger and more discrete SKAP1 clusters that were concentrated in the peripheral contact band of the contact region Fig. 1b, left lower panels). LAT-cherry and SKAP1-GFP clusters overlapped in this region (Fig. 1b, see yellow dots). These data confirmed that LFA-1 cross-linking promoted SKAP1-LAT proximity or clusters, which appeared primarily in the peripheral contact band, an area known for the localization of LFA-1.

We next assessed biochemically whether LAT could bind to SKAP1 in response to LFA-1 cross-linking (Fig. 1c). DC27.10 mouse T cells were cross-linked with antibody followed by precipitation with anti-SKAP1 or LAT. Membrane and cytosolic fractions were purified by cell disruption and centrifugation, as described in the Methods^37^. Anti-SKAP1 co-precipitated LAT in response to anti-LFA-1 alone and anti-CD3/LFA-1 ligation from membranes (Fig. 1c, upper panels, lanes 4 and 5). Anti-LFA-1 induced more SKAP1/LAT binding than anti-CD3 in over 8 experiments (Fig. 1c, lanes 4 versus 3). Anti-CD3/LFA-1 cooperated to produce the greatest SKAP1/LAT binding (Fig. 1c, lane 5), consistent with PLA analysis (Fig. 1a). No co-precipitated SLP-76 was observed in the anti-SKAP1 IPs. Similarly, anti-LAT co-precipitated more SKAP1 in response to anti-LFA-1/CD3, more than seen with anti-CD3 ligation from membranes (Fig. 1c, lower panel, lane 6 versus 5).

Further, the cross-linking of LFA-1 by an incubation of cells with plate immobilized ICAM1-Fc induced LAT-SKAP1 complexes as seen by the co-precipitation of LAT with anti-SKAP1 from the membrane fraction (Fig. 1d, lanes 8, 9). No association was seen in precipitates from the cytosolic fractions (Fig. 1d, lanes 3–5). Neither GADS nor SLP-76 was observed in the co-precipitates.

Plate immobilized ICAM1-Fc alone or in combination with soluble anti-CD3 also induced SKAP-1-LAT binding in peripheral blood human primary T cells (Fig. 1e). Anti-SKAP1 was observed to co-precipitate LAT from the membrane fraction of cells ligated by ICAM1-Fc over 5–20 min (Fig. 1e, upper panel, lanes 3–5) and by anti-CD3/LFA-1 (Fig. 1e, lanes 6–8). The increased level of LAT associated SKAP1 correlated with the increased presence of SKAP1 in the membrane fraction of cells. We previously showed that activation signals induce the translocation of SKAP1 to the membranes of T cells^37^,^38^. Neither GADs nor SLP-76 was observed in the co-precipitates. Collectively, these data showed that the cross-linking of LFA-1 alone or together with anti-CD3 induced the association of SKAP1 with LAT in mouse and human T cells.

### LAT-SLP-76 and LAT-SKAP1 are distinct complexes

**Figure 2:**
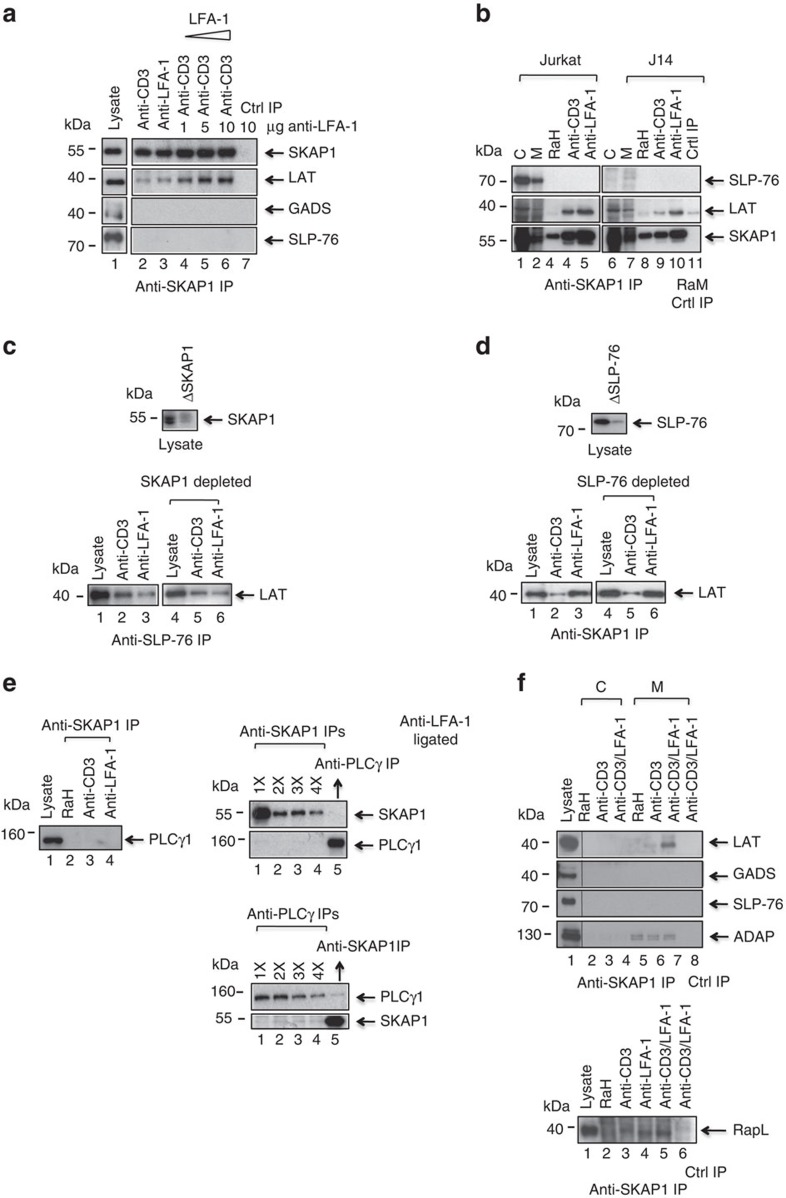
Anti-LFA-1 induced SKAP1-LAT forms independently of SLP-76. (**a**) Titration of anti-LFA-1 increases LAT-SKAP1 binding. DC27.10 T cells were ligated with anti-CD3 (2 μg ml^−1^) in the presence of different concentrations of anti-LFA-1 followed by precipitation with anti-SKAP1 and blotting with anti-SKAP1, LAT, GADS and SLP-76 (*n*=3). (**b**) SKAP1 binds to LAT in SLP-76 deficient J14 Jurkat cells. Jurkat and J14 Jurkat T-cells were ligated with anti-CD3 (1 μg ml^−1^) and/or anti-LFA-1 (1 μg ml^−1^) for 5 min followed by membrane preparation, anti-SKAP1 precipitation and blotting for anti-SLP-76, anti-LAT or anti-SKAP1 (*n*=3). (**c**) SLP-76 co-precipitates LAT from SKAP1-depleted cell lysates. Upper panel: Blot showing anti-SKAP1 depletion of SKAP1. Jurkat cells ligated with anti-CD3 and/or anti-LFA-1 for 5 min followed by detergent solubilization and serial depletion of lysates with anti-SKAP1 (5 times), anti-SLP-76 precipitation and anti-LAT blotting (*n*=3). (**d**) SKAP1 co-precipitates LAT from SLP-76-depleted cell lysates. Upper panel: Blot showing anti-SLP-76 depletion of SLP-76. Jurkat T-cells were ligated with anti-CD3 and/or anti-LFA-1 for 5 min followed by detergent solubilization and serial depletion of lysates with anti-SLP-76 (five times), anti-SKAP1 precipitation and anti-LAT blotting (*n*=3). (**e**) SKAP1 does not associate with PLCγ1. Jurkat T-cells were ligated, detergent solubilized and precipitated by anti-SKAP1. Western blotting was then conducted with anti-PLCγ1 (left panel). Upper right panel: cells were cross-linked with anti-LFA-1 and sequentially precipitated with anti-SKAP1 and each precipitate was subjected to western blotting with anti-SKAP1 (lanes 1–4) or anti-PLCγ1 (lane 5). Lower right panel: Cells were depleted sequentially with anti-PLCγ1 and each were the blotted with anti- PLCγ1 (lanes 1–4) or anti-SKAP1 (lane 5) (*n*=3). (**f**) SKAP1 associates with ADAP and RapL. Jurkat T-cells were ligated with antibodies as shown, were precipitated with anti-SKAP1 and subjected to blotting with anti-LAT, GADs, SLP-76 and ADAP (upper panels). The same cells were ligated precipitated as above but then subjected to blotting with anti-RapL (*n*=3).

The absence of SLP-76 or GADs in anti-SKAP1 precipitates suggested that LAT-SKAP1 and LAT-GADS-SLP-76 were distinct complexes. To obtain more information, we next ran a titration with increasing anti-LFA-1 concentrations in the presence of a fixed concentration of anti-CD3 (2 μg ml^−1^) (Fig. 2a, lanes 4–6). This showed that greater levels of LFA-1 cross-linking increased the level of LAT co-precipitated by anti-SKAP1. Further, we next assessed whether the formation of LAT-SKAP1 complexes required SLP-76 expression, and vice versa (Fig. 2b). SLP-76-deficient J14 cells were ligated with anti-LFA-1 followed by anti-SKAP1 precipitation. Anti-SKAP1 co-precipitated LAT similarly from LFA-1-ligated wild-type and SLP-76-deficient J14 cells (Fig. 2b, middle panel, lanes 5 and 10).

Further, depletion analysis of cell lysates was also conducted (Fig. 2c). Anti-SKAP1 depleted SKAP1 from lysates by carrying out serial precipitations of the lysate (5 × ) prior to precipitating with anti-SLP-76. Anti-SKAP1 depletion reduced the presence of SKAP1 in the cell lysate (Fig. 2c, upper panel). Anti-SLP-76 co-precipitated LAT from depleted and non-depleted lysates of cells stimulated with anti-CD3 (Fig. 2c, lanes 2 and 5). Serial precipitations (6 × ) with anti-SLP-76 were carried out to deplete lysates of SLP-76 (Fig. 2d, upper panel). We observed that anti-SKAP1 co-precipitated equal levels of LAT from SLP-76 non-depleted and depleted lysates from cells ligated with anti-LFA-1 (Fig. 2d, lower panel, lane 3 and 6). Anti-SKAP1 co-precipitated more LAT from anti-LFA-1 than anti-CD3 cross-linked cells. Taken together, these data indicated that there are distinct complexes of LAT-SLP-76 and LAT-SKAP1 in T cells.

Previous studies have shown that ant-CD3 induction of phospholipase Cγ-1 (PLCγ1) binding to pY-132 on LAT is key to the generation of activation signals by the adaptor^59^. However, anti-SKAP1 failed to co-precipitate PLCγ1 as seen in lysates from cells that been ligated for 10 min with anti-LFA-1 or anti-CD3 and blotted with anti- PLCγ1 (Fig. 2e, left panel, lanes 4 and 3, respectively). Similarly, anti-PLCγ1 blotting of lysates sequentially depleted of SKAP1 (1–4 × ) failed to show a co-precipitated band (Fig. 2e, upper right panel). As a control, PLCγ1 was detected in anti-PLCγ1 precipitates (lane 5). Conversely, anti-SKAP1 blotting of lysates that had been sequentially depleted of PLCγ1 also failed to show a band (Fig. 2e, lower right panel).

At last, as an additional observation, anti-SKAP1 co-precipitated ADAP from resting, anti-CD3 and LFA-1 ligated cells (Fig. 2f, upper panel)^18^,^36^,^37^. Further, anti-SKAP1 co-precipitated RapL from anti-CD3 and LFA-1-ligated cells cells^37^,^38^ (Fig. 2f, lower panel).

### Adaptor GRB2 binds to SKAP1

**Figure 3:**
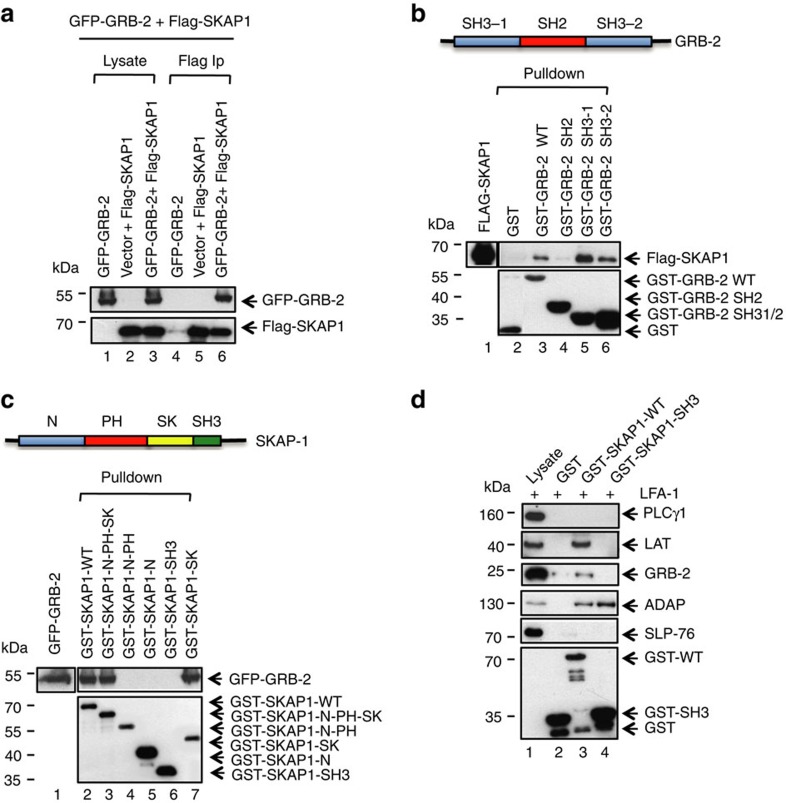
GRB2 binds via its SH3 domains to SKAP1. (**a**) GRB2 binds to SKAP1. 293T cells were transfected with GFP-GRB2, FLAG-SKAP1 or both, followed by anti-Flag precipitation and blotting with anti-GFP or anti-Flag. Lanes 1–3: cell lysates; lanes 4–6: anti-Flag precipitations (*n*=3). (**b**) GRB2 SH3-1 and 2 domains bind to SKAP1. 293T cells were transfected with FLAG-SKAP1, followed by detergent solubilization of cells and GST pull-down with versions of GST-tagged GRB-2 constructs and blotting with anti-Flag or anti-GST (*n*=3). (**c**) GRB2 SH3 domains bind to the SK region of SKAP1. 293T cells were transfected with GFP-tagged GRB2 followed by detergent solubilization of cells, and GST pull-down with various versions of GST-SKAP1 (GST-SKAP1-WT1; GST-SKAP1-N-PH-SK; GST-SKAP1-N-PH; GST-SKAP1-PH; GST-SKAP1-SH3; GST-SKAP1-SK) and blotting with anti-GFP or anti-GST (*n*=3). (**d**) GST-SKAP1 co-precipitates LAT, GRB-2 and ADAP from Jurkat T-cells. Jurkat cells were ligated with anti-LFA-1 for 5 min. followed by lysis of cells, pull down with GST, GST-SKAP1 WT or GST-SKAP SH3 domain followed by blotting with anti- PLCγ1, anti-LAT, anti-GRB-2, anti-ADAP and anti-SLP-76 and anti-GST. GST-SKAP WT pulled down LAT, GRB-2, ADAP, but neither PLCγ1 nor SLP-76 (*n*=3).

We next assessed whether SKAP1 binds to the LAT binding partners, GRB-2, GADs and PLCγ1 (Fig. 3, Supplementary Fig. 1). Flag-tagged SKAP1 was co-expressed with GFP-GRB-2 in 293T cells followed by anti-SKAP1 precipitation and blotting. SKAP1 precipitated GRB-2 as detected by anti-GFP blotting (Fig. 3a, lane 6). Western blotting of lysates with anti-Flag confirmed the expression of SKAP1 (Fig. 3a, lower panel, lanes 1–3). By contrast, Flag-tagged SKAP1 failed to co-precipitate either GADS or PLCγ1 (Supplementary Fig. 1a, lane 6). These data showed that GRB-2, but neither GADs nor PLCγ1, bind to SKAP1.

To determine which GRB-2 domain binds to SKAP1, GST-GRB-2 SH2 and GST-GRB-2 SH3 domains were used in a GST-pull-down assay with co-expressed SKAP1 followed by blotting with anti-SKAP1. From this, the two GST-GRB2-SH3 domains were seen to precipitate SKAP1 (Fig. 3b, lanes 5, 6), whereas the GST-SH2 domain failed to precipitate the adaptor (Fig. 3b, lane 4). The *N*-terminal SH3-1 domain precipitated more effectively than the SH3-2 domain (Fig. 3b, lane 5 versus 6). These data indicated that the SH3 domains of GRB-2 mediate the binding to SKAP1.

To assess the region in SKAP1 that is recognized by the GRB2 SH3 domain, GST-SKAP1 wild-type and sub-domains were used in a pull-down assay with GFP-GRB2 followed by blotting with anti-GRB2. GST-SKAP1 WT, GST-SKAP1 N-PH-SK and GST-SKAP1-SK precipitated GFP-GRB2 (Fig. 3c, lanes 2, 3, 7, respectively). By contrast, the GST-SKAP1 N-PH domain, GST-SKAP1 N-domain and GST-SKAP1 SH3 domain failed to precipitate the protein GRB2 (Fig. 3c, lanes 4, 5, 6, respectively). From this, it was evident that the SK region was recognized by GRB-2.

At last, for confirmation of the interaction in Jurkat T cells, GST-SKAP1 fusion proteins were used in pull-down assays to precipitate antigen from anti-LFA-1 ligated Jurkat T cells. GST SKAP1 WT precipitated LAT, GRB-2 and ADAP from lysates (Fig. 3d, lane 3). Neither PLCγ1 nor SLP-76 was co-precipitated. As a control, the GST-SKAP1 SH3 domain failed to precipitate GRB2, but did precipitate ADAP (Fig. 3d, lane 4), as we and others have reported^35^,^36^.

### LFA-1 recruits FAK-1 to selectively phosphorylate LAT Y-171

**Figure 4:**
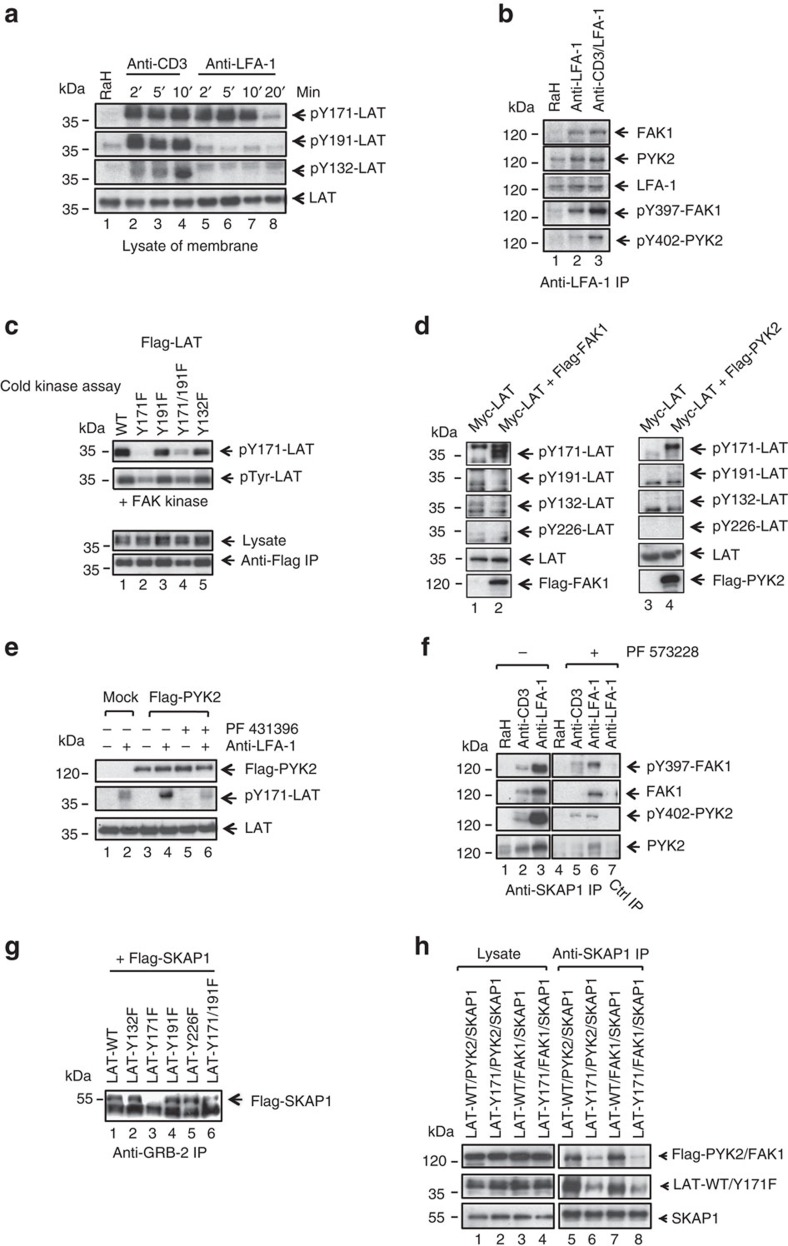
LFA-1, FAK1 and PYK2 phosphorylate restricted site Y-171 on LAT. (**a**) Anti-LFA-1 cross-linking selectively phosphorylates LAT Y-171. Jurkat cells were ligated with anti-CD3 or anti-LFA-1/CD3 for 2–20 min, followed by membrane preparation and blotting for pY-171-LAT, pY-191-LAT, pY-132-LAT and with anti-LAT (*n*=3). (**b**) LFA-1 associates with and activates FAK1 and PYK2. Jurkat cells were ligated with anti-CD3 and/or anti-LFA-1 prior to anti-LFA-1 precipitation and blotting with anti-LFA-1, anti-pY-397-FAK, anti-pY-402-PYK2, anti-FAK1 and anti-PYK2 (*n*=3). (**c**) FAK1 *in vitro* kinase phosphorylation of LAT is dependent on the Y-171 residue. 293T cells were transfected with Flag-tagged LAT-mutants, precipitated with anti-Flag and subjected to a cold *in vitro* kinase assay with recombinant FAK kinase (Millipore), followed by blotting with ant-pY-171-LAT, anti-pTyr (4610) and anti-Flag (*n*=3). (**d**) Co-expression of LAT with FAK1 or PYK2 selectively phosphorylates Y-171. 293T cells were transfected with Myc-tagged LAT and Flag-tagged FAK1 or PYK2 and subjected to blotting with anti-phospho-specific antibodies against LAT. Left panel: Myc-LAT and Flag-FAK1; right panel: Myc-LAT and Flag-PYK2 (*n*=3). (**e**) Anti-LFA-1 induced pY-171 is inhibited by FAK1/PYK2 inhibitor PF 431396. Jurkat T-cells transfected with either vector control (mock) or Flag-PYK2 were ligated with anti-LFA-1 (1 μg ml^−1^) in the absence or presence of FAK1/PYK2 inhibitor PF 431396, followed by blotting with anti-Flag, anti-pY-171-LAT or anti-LAT (*n*=3). (**f**) Anti-LFA-1 induced SKAP1-FAK1/PYK2 complex is inhibited by FAK1/PYK2 inhibitor PF573228. Jurkat T cells were ligated with rabbit anti-hamster (control), anti-CD3 (1 μg ml^−1^) or anti-LFA-1 (1 μg ml^−1^) in the absence or presence of PF573228, followed by precipitation with anti-SKAP1 and blotting with anti-FAK1, anti-pY-397-FAK, anti-PYK2 or pY-402-PYK2 (*n*=3). (**g**) Anti-LFA-1 induced LAT-GRB2-SKAP1 depends on LAT Y-171 site. 293T cells were transfected with Flag-SKAP1 and various Myc-tagged LAT mutants (LAT-WT; LAT-Y132F; Lat-Y171F; LAT-Y191F; LAT-Y226F: LAT-Y171/191F) followed by anti-GRB-2 precipitation and blotting with anti-Flag. GRB-2 is endogenously expressed in 293T cells (*n*=3). (**h**) Anti-SKAP1 co-precipitates FAK1/PYK2 and LAT dependent on LAT-Y-171. 293T cells were transfected with LAT-WT, PYK2 and SKAP1, LAT-Y-171F, PYK2 and SKAP1, LAT-WT, FAK1 and SKAP1 or LAT-Y-171F, FAK1 and SKAP1 followed by precipitation with anti-SKAP1 and blotting with anti-FLAG, anti-LAT or anti-SKAP1. Lysate (lanes 1–4) anti-SKAP1 IP (lanes 5–8) (*n*=3).

Next, we assessed whether LFA-1 cross-linking could induce LAT phosphorylation (Fig. 4a). DC27.10 T cells were incubated with anti-CD3 or anti-LFA-1 for 5 min followed by membrane purification and blotting with anti-phospho-specific LAT antibodies. As previously described^59^, anti-CD3 induced the phosphorylation of LAT at residues Y-171, Y-191 and Y-132 over 2–10 min (Fig. 4a, lanes 2–4 versus 1). Remarkably, anti-LFA-1 phosphorylated LAT at only single site Y-171 (Fig. 4a, lanes 5–8). The level of Y-171 phosphorylation was similar, or greater, than seen with anti-CD3 (*n*=7). Neither Y-191 or Y-132 was phosphorylated. As a control, anti-LAT blotting showed the expression of equal levels of protein in the solubilized membrane fractions. These data showed the novel finding that LFA-1 phosphorylated LAT at a restricted Y-171 residue.

This phosphorylation of a single site suggested that LFA-1 uses a kinase distinct from ZAP-70. FAK1 and PYK2 can associate with focal adhesions where auto-phosphorylation is an essential step for activation^47^. We therefore assessed whether these kinases associated with LFA-1 and mediated phosphorylation (Fig. 4b). Jurkat T cells were ligated with anti-LFA-1 or anti-CD3/LFA-1 for 5 min followed by anti-LFA-1 precipitation and blotting. Anti-LFA-1 precipitation from cells cross-linked with anti-LFA-1 or anti-CD3/LFA-1 showed the increased presence of FAK1 and PYK2 (Fig. 4b, lanes 2, 3, upper panels). Although no LFA-1 associated FAK1 was found in resting cells (that is, rabbit control anti-mouse) (Fig. 4b, lane 1), associated PY-2 seen in resting cells was increased by LFA-1 ligation (Fig. 4b, lanes 2, 3 versus 1). Importantly, LFA-1 ligation also activated the associated FAK1 and PYK2 kinases, as seen with antibodies to the auto-phosphorylation sites (that is, FAK at Y-397 or PYK2 at Y-402) (Fig. 4b, lane 2) or LFA-1/CD3 (Fig. 4b, lane 3). These data showed that LFA-1 cross-linking induces the association and activation of FAK1 and PYK2 kinases.

We next used a cold *in vitro* kinase assay to assess whether FAK1 directly phosphorylated Y-171, (Fig. 4c). 293T cells were transfected with various mutants of Flag-tagged LAT. Anti-Flag was used to precipitate LAT followed by an *in vitro* kinase assay in the presence of exogenous added recombinant FAK1 and non-radioactive ATP followed by blotting with an anti-phosphotyrosine (4G10). In this, wild-type LAT, Y-191F and the Y-132F mutants were phosphorylated by FAK1 (Fig. 4c, lanes 1, 3 and 5, respectively). However, Y-171F and Y-171/191-F mutants showed a markedly reduced signal (Fig. 4c, lanes 2, 4). Anti-Flag blotting confirmed the equal expression and precipitation of WT LAT and mutants. These data showed that Y-171 was the preferred phosphorylation site of FAK1 in an *in vitro* kinase assay.

We also co-transfected 293T cells with Myc-tagged LAT and either Flag-tagged FAK1, or PYK2, followed by precipitation and blotting with anti-phospho-LAT specific antibodies (Fig. 4d). Remarkably, again, FAK1 phosphorylated LAT on Y-171, but not on Y-191, Y-132 or Y-226 (Fig. 4d, lane 2 versus 1). The expression of related Flag-PYK2 also phosphorylated LAT on Y-171 but not on the other sites (Fig. 4d, lane 4 versus 3).

Jurkat T cells were also transfected with Flag-PYK2 followed by cross-linking with anti-LFA-1 in the presence or absence of the FAK1/PYK2 inhibitor PF 431396. Anti-LFA-1 cross-linking induced Y-171 phosphorylation in mock-transfected cells (Fig. 4e, lane 2 versus 1), and this was further increased by PYK2 transfection (Fig. 4e, lane 4). PF431396 inhibited the anti-LFA-1 induced phosphorylation of Y-171 (Fig. 4e, lane 6). These data showed that PYK2 expression co-operated with LFA-1 cross-linking to induce the phosphorylation of the Y-171 site.

Similarly, activated FAK1/PYK2 were found associated with SKAP1. Jurkat cells were ligated for 5 min in the presence of FAK1/PYK2 inhibitor PF 573228 followed by precipitation with anti-SKAP1. Anti-LFA-1 induced the binding of FAK-1 and PYK2 to SKAP1 (Fig. 4f, lanes 3 versus 1). Second, SKAP1-associated FAK1 and PYK2 were active as seen by anti-FAK pY-397 or anti-PYK2 pY-402 blotting (Fig. 4f, lane 3). Third, the presence of associated FAK1 and PYK2 was reduced by inhibition of kinase activity (Fig. 4f, lane 6 versus 3). Anti-FAK pY-397 or anti-PYK2 pY-402 blotting confirmed the inhibition of kinase activity by the drug. These data confirmed that anti- LFA-1 cross-linking induced the binding of FAK1/PYK2 to SKAP1, an event dependent on increased FAK1/PYK2 phosphorylation and activity. By contrast, anti-CD3 increased the FAK1/PYK2 signal, but at a level lower than seen with anti-LFA-1 (Fig. 4f, lane 2).

To assess whether the association of SKAP1 with endogenous GRB-2 was dependent on binding to the Y171 site on LAT, LAT wild-type or mutants were co-expressed with SKAP1 in 293T cells, followed by anti-GRB2 precipitation and blotting with anti-SKAP1. Unlike LAT and SKAP1, GRB2 is endogenously expressed in 293T cells. Anti-GRB-2 co-precipitated SKAP1 from cells expressing wild-type and the Y-132, Y-191, Y-226 mutants (Fig. 4g, lanes 1, 2, 4, 5, respectively). By contrast, the anti-GRB-2 co-precipitated SKAP1 was greatly reduced in precipitates from cells expressing Y-171, or Y-171/Y-191 (Fig. 4g, lanes 3, 6). GRB-2 binding to SKAP1 (that is, SH3 domain) was therefore influenced by GRB-2 binding to LAT (that is, SH2 domain).

To further determine whether LAT and the LAT pY-171 site was needed for anti-SKAP1 co-precipitation of the kinases, various combinations of Flag-FAK, Flag-PYK2, SKAP1 and LAT were co-expressed in 293T cells and assessed for binding (Fig. 4h). Anti-SKAP1 co-precipitated LAT and Flag-FAK1 or Flag-PYK2 from cells co-transfected with WT LAT, SKAP1 and Flag-PYK2 or Flag-FAK1 (Fig. 4h, lanes 5 and 7). By contrast, greatly reduced when LAT-pY171 and Flag-PYK-2 or Flag-FAK was co-precipitated by anti-SKAP1 (Fig. 4h, lanes 6 and 8). These observations showed that SKAP1 binding to FAK1 and PYK2 requires the presence of binding sites on LAT at Y-171.

### LFA-1 cross-linking is needed to activate FAK1 and LAT pY-171

**Figure 5:**
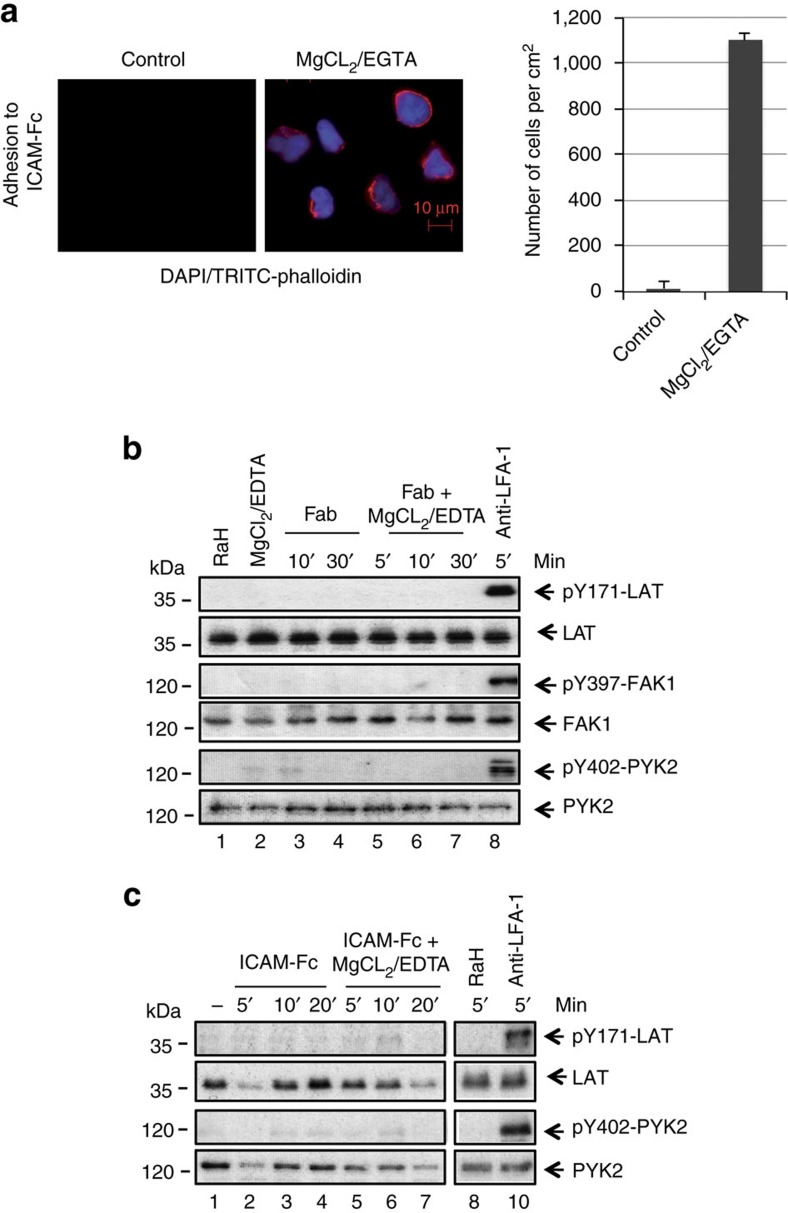
LFA-1 cross-linking needed for pY-171 and FAK auto-phosphorylation. (**a**) MgCl_2_/EDTA induced the adhesion of T cells to ICAM-1 on plates. Left panels: images of T cells bound to ICAM-Fc (left: untreated controls; right: MgCl_2_/EDTA treated cells stained with DAP1 and TRITC-phalloidin). Right panel: histogram showing MgCl_2_/EDTA induced increases in T-cell binding to ICAM1-Fc (*n*=3). (**b**) Antibody cross-linking is needed to induce pY-171 and FAK/PYK2 auto-phosphorylation. Jurkat cells were either incubated with soluble anti-LFA-1 Fab’ or Fab’ plus MgCl_2_/EDTA or full sized anti-LFA-1 for various times followed by blotting with anti-pY171- LAT, anti-LAT, anti-pY-397-FAK, anti-FAK or anti-pY-402-PYK2 and anti-PYK2. Assay was performed in the absence of ICAM1 on plates (*n*=3). (**c**) Mono-valent ICAM1 failed to induce pY-171 and FAK/PYK2 auto-phosphorylation. Jurkat cells were either incubated with soluble ICAM1-Fc or ICAM1-Fc plus MgCl_2_/EDTA or bivalent anti-LFA-1 for various times followed by blotting with anti-pY171- LAT, anti-LAT, anti-pY-402 PYK2 and anti-PYK2. Assay was performed in the absence of ICAM1 on plates (*n*=3).

We next assessed whether increased LFA-1 affinity mediated binding could activate FAK1/PYK2 and LAT -pY-171, or whether receptor cross-linking was required. The combination of 5 mM MgCl^2+^ and 1 mM EGTA buffer is known to induce conformational changes for high-affinity LFA-1 without receptor cross-linking^22^,^60^. To confirm the effectiveness of MgCl^2+/^EGTA, Jurkat cells were incubated with the reagents for 15 min and assess for adhesion to immobilized ICAM-Fc on plates (Fig. 5a). This showed a marked increase in the number of cells bound to plates (that is, 1,800 versus 10 cells per cm^−2^), thereby confirming affinity activation.

Given this, we next incubated Jurkat cells with MgCl_2_/EDTA, soluble anti-LFA-1 Fab’, soluble Fab’ plus MgCl_2_/EDTA or soluble anti-LFA-1 for various times followed by blotting with anti-pY171 LAT, anti-LAT, anti-pY-397-FAK, anti-FAK or anti-pY-402-PYK2 and anti-PYK2 (Fig. 5b). The assay was performed in the absence of ICAM1 on plates. Despite affinity activation, incubation with MgCl^2+^/EGTA induced no LAT pY-171, pY397-FAK1 or pY401-PYK2 phosphorylation (Fig. 5b, lanes 2 versus 1). Further, the engagement of LFA-1 with mono-valent Fab’ anti-LFA-1 failed to induce phosphorylation, either alone or in the presence of MgCl^2+/^EGTA (Fig. 5b, lanes 3–7). In contrast, the cross-linking of LFA-1 on the same cells with bivalent anti-LFA-1 induced the phosphorylation of LAT Y-171, Y397-FAK1 or Y401-PYK2 (Fig. 5b, lane 8). Similarly, incubation of cells with soluble mono-valent ICAM1-Fc alone, or in combination with ICAM1-Fc MgCl^2+^/EGTA over 5–20 min did not induce phosphorylation of Y-171 LAT or pY401-PYK2 (Fig. 5c, lanes 2–7). As a control, bivalent anti-LFA-1 cross-linking induced pY-171 LAT (Fig. 5c, lane 10). Together, this data showed that the activation of FAK1 and PYK2 and phosphorylation of LAT Y-171 required LFA-1 cross-linking.

### FAK1 limits T-cell dwell times with DCs

**Figure 6:**
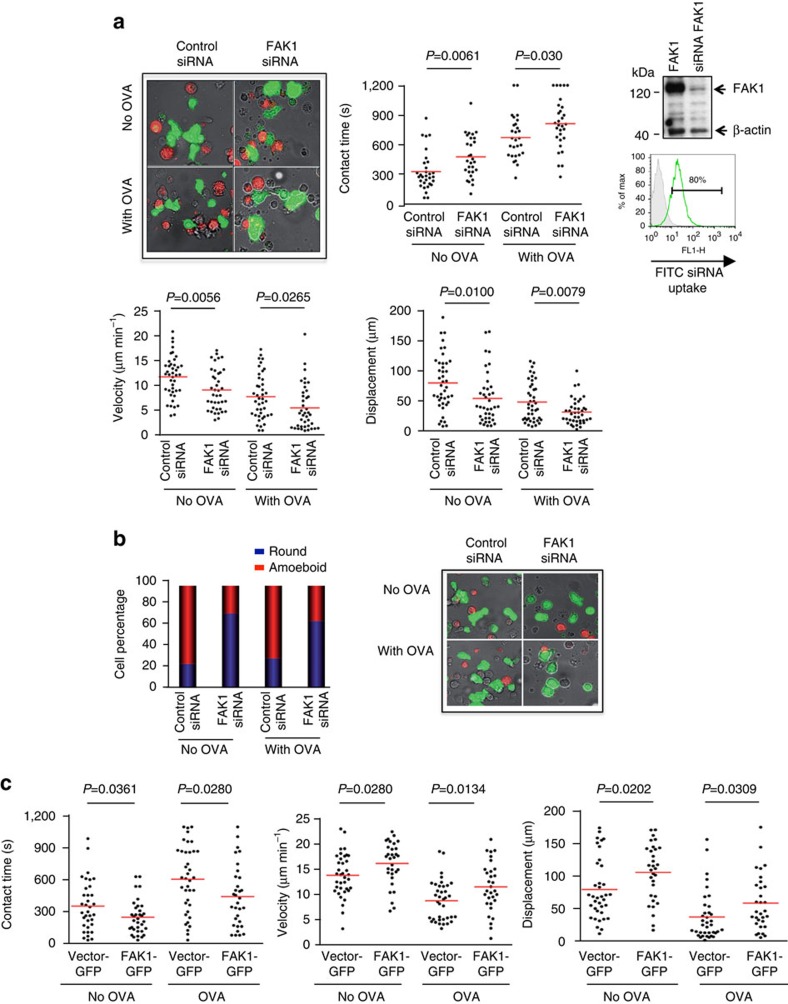
FAK1 reduces T cell contact with DCs. (**a**) FAK1 siRNA increased contact times while reducing velocity. DO11.10 T cells were pre-stimulated for 24 h, resting for 12 h and transfected with siRNA for FAK1. Mature DCs were labelled with SNARF-1 and pre-incubated with OVA peptide (DC-OVA) prior to incubation with T cells on LN slices, as described^37^,^61^. DO11.10 T cells were labelled with CFSE and tracked for migration on LN slices. Upper left panel: image example of T cells interacting with DCs; upper middle panel: dot plots showing that FAK1 siRNA increases T-cell dwell times with DCs; upper right panel: anti-FAK1 blot showing reduction in FAK1 expression (lower: FITC siRNA uptake as shown by flow cytometry); lower left panel: dot plot showing that FAK1 siRNA reduces the velocity of T cells; lower right panel: dot plots showing that FAK1 siRNA reduces the displacement of T cells (*n*=3). (**b**) siRNA knockdown of FAK1 decreased the amoeboid morphology of DO11.10 T cells. siRNA expression increased the percentage of cells with rounded morphology while decreasing those with an amoeboid shape (4 × length relative to width). Left panel: histogram of ratio of rounded versus amoeboid cells; right panels: images of cells (*n*=2). (**c**) FAK1 overexpression decreases dwell times with increased motility and displacement. DO11.10 T cells were prepared as above and transfected with GFP-FAK1. Mature DCs were labelled with SNARF-1 and pre-incubated with OVA peptide (DC-OVA) prior to incubation with T cells. Left panel: dot plot showing that FAK1 expression reduces contact times; middle panel: dot plot showing that FAK1 expression increases velocity; right panel: dot plot showing that FAK1 expression increases displacement (*n*=3). *P* values were calculated by one-way analysis of variance (ANOVA) between groups followed by a series of *t*-tests.

We next assessed whether FAK1 and LAT-Y171 influenced the dwell times of T cells with DCs. DO11.10 CD4+ T cells were transfected with FAK1 siRNA followed by a measure of the dwell times with DCs, as described^37^,^61^ (Fig. 6). Mature DCs were labelled with SNARF-1 and pre-incubated with OVA peptide (DC-OVA) prior to incubation with T cells^61^. FITC-siRNA electroporation showed an 80% siRNA uptake by T cells and reduced expression of FAK1 by anti-FAK1 blotting (Fig. 6a, right panels). These T cells showed significantly longer contact times with DCs than T cells transfected with the siRNA scrambled control. FAK1 siRNA increased the mean contact time of T cells from 675 to 814 s in the presence of OVA peptide, and from 334 to 482 s in the absence of peptide (Fig. 6a, left and middle upper panels). Antigen independent conjugation between T cells and DCs has been previously described. Concurrently, we observed a reduction in T-cell motility and displacement owing to FAK1 siRNA (Fig. 6a, lower right and left panels). In the presence of OVA peptide, T cells with FAK siRNA showed a mean velocity of 6 μm min^−1^ compared with 8.6 μm min^−1^ for scrambled, whereas velocity in the absence of antigen increased from 10 μm min^−1^ relative to 13 μm min^−1^ for the scrambled control. Similarly, displacement (distance from the site of origin) decreased with FAK1 siRNA from 48 to 32 μm in the presence of peptide and from 80 to 54 μm in its absence.

Consistent with this, FAK1 siRNA T cells were also more rounded and less polarized (that is, less amoeboid) in the presence and absence of OVA peptide relative to cells expressing control siRNA (as defined as 1.4/1.0 length versus width) (Fig. 6b; right panel and images).

Conversely, FAK1-GFP transfection of DO11.10 CD4+ T cells reduced contact times with DCs (Fig. 6c, left panel). In the presence of OVA peptide, we found that FAK1-GFP transfected cells showed a mean contact time of 486.3 relative to 664.2 s for vector-GFP transfected control cells. In the absence of antigen, the cells showed a mean reduced dwell time of 275 compared with 389 s for vector-GFP. At the same time, mean motility was significantly increased from 8.9–11.7 s in the presence of antigen and 14–16.4 s in its absence of OVA peptide (Fig. 6c, middle panel). Displacement was also increased from 41 to 65 μm in the presence of antigen and 88 to 117 μm in the absence of OVA peptide (Fig. 6c, right panel). These observations show that FAK1 in T cells reduces contact times between T cells and DCs.

### FAK limitation on T-cell dwell times depends on LAT Y-171

**Figure 7:**
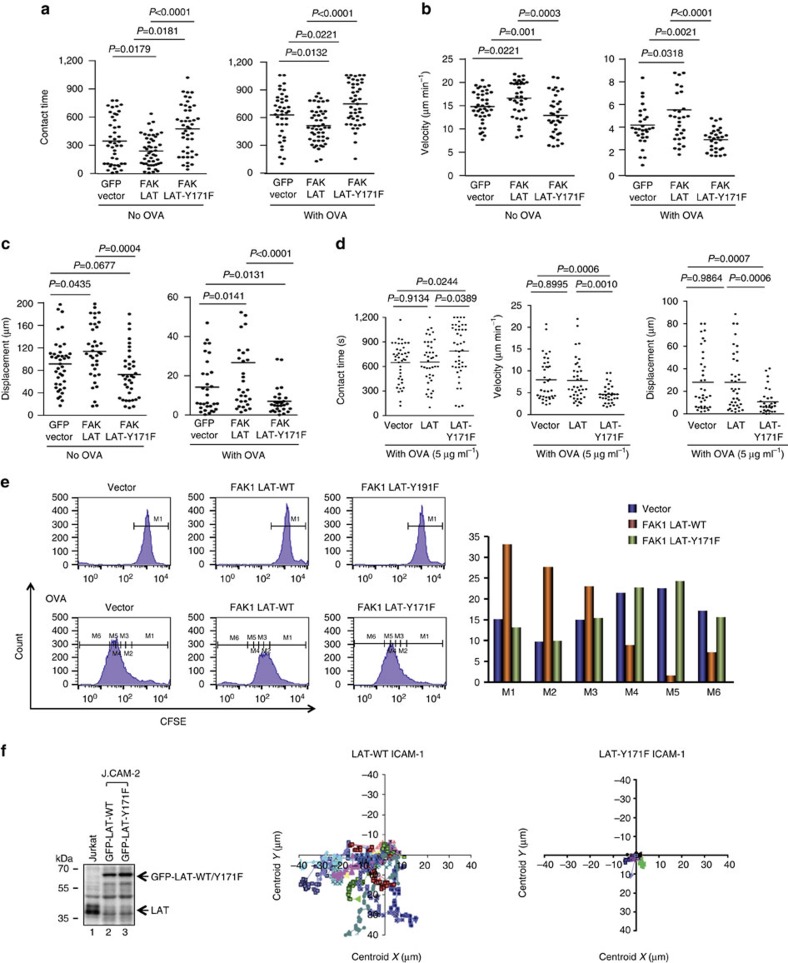
FAK1 reduces T-cell contact times with DCs, an effect dependent on the LAT Y-171. (**a**) Dot plots showing that LAT-Y-171F expression reverses FAK1 inhibition of T-cell dwell times. DO11.10 T cells cells were prepared as above and co-transfected with FAK1 and LAT or LAT-Y171F. Mature DCs were labelled with SNARF-1 and pre-incubated with OVA peptide (DC-OVA) prior to incubation with T cells. (**b**) Dot plots showing that LAT-Y-171F expression reverses FAK1 increase in T-cell velocity. DO11.10 T cells were prepared as above. (**c**) Dot plots showing that LAT-Y-171F expression reverses FAK1 increase in T-cell displacement. DO11.10 T cells were prepared as above. (**d**) LAT-Y-171F expression alone increases T-cell dwell times. LAT-Y171F increases T-cell contact (left panel); LAT-Y171F decreases T-cell motility (middle panel); LAT-Y171F decreases T-cell displacement (middle panel). D011.10 T cells were prepared as above and co-transfected with LAT-Y171F prior to a measure of T-cell contact with DCs. (**e**) LAT-Y171F expression reverses FAK1 inhibition of DO11.10T-cell proliferation in response to OVA peptide. DO11.10 T cells were prepared as above except that they were pre-labelled with CFSE. Left panels: FACs histograms of CFSE labelling; right histogram: panels showing the inhibition of T-cell cycling by FAK1 LAT-WT (gold columns showing most cells in M1–M3, whereas FAK1 LAT Y-171F shows the increased presence of cells in M3-M6 (*n*=4). (**f**) LAT-Y-171F expression blocks T-cell motility/migration on ICAM1-Fc coated plates. J14 Jurkat cells were transfected with LAT or LAT-Y-171F and tracked for migration. Left panel: immunoblots of lysates transfected with LAT or LAT-Y-171F; middle panel: tracking profiles of LAT transfected J14 T cells; right panel: tracking profiles of LAT-Y-171F transfected J14 T cells (*n*=3). *P* values were calculated by one-way analysis of variance (ANOVA) between groups followed by a series of *t*-tests.

Given the phosphorylation of LAT Y-171 by FAK1, we next asked whether the FAK1 induced reduction in T-cell conjugation was dependent on LAT Y-171 (Fig. 7). In this case, DO11.10 CD4+ T cells were co-transfected with FAK1-GFP and wild-type LAT or LAT-Y171F followed by a measurement of dwell times. In the presence of OVA peptide, the combination of FAK-GFP and LAT reduced the mean contact times from 751.1 to 583.3 s, and in the absence of antigen, from 396 to 275 s (Fig. 7a). Conversely, the combination of FAK1-GFP and LAT increased motility and displacement (Fig. 7b,c).

Significantly, by contrast, the co-expression of the LAT-Y171F mutant completely reversed the reduced dwell effect of FAK1-GFP on conjugation times (Fig. 7a, upper left panels). It even increased the mean contact from 715 to 851 s in the presence of OVA and from 396 to 544 s in the absence of OVA peptide (Fig. 7a, upper left panels). This increase in contact time was even higher than seen in the GFP vector control cells. Similarly, the expression of LAT-Y171F blocked the ability of FAK1 to increase the mean velocity and displacement of cells (Fig. 7b,c, right and lower panels). These observations showed that the ability of FAK1 to limit T-cell contact with DCs was dependent on LAT Y-171, the site phosphorylated by FAK1.

Similar results were obtained when LAT-Y171F was expressed in T cells in the absence of co-transfected FAK1 (Fig. 7d). In the absence of overexpressed FAK1, the effect of the wild-type LAT was less apparent, however, the expression of LAT-Y171F increased contact times (620–850 s) (left panel) concurrent with a reduced velocity (7.7–4.5 μm min^−1^) (Fig. 7d, middle panel) and displacement (27–12 μm) (Fig. 7d, right panel). These observations showed that LAT-Y171F was effective in modulating conjugation in the presence of endogenous FAK1.

This modulation of dwell times by FAK1 and LAT-Y171F was also reflected in the proliferative response of DO11.10T cells to OVA peptide (Fig. 7e). DO11.10T cells were labelled with carboxyfluorescein diacetate succinimidyl ester, incubated with DCs plus OVA peptide and assessed for cell division by flow cytometry at day 6. Although vector-transfected cells under cell division can be seen as the presence of M2–M6 cell divisions, transfection of FAK1 and WT LAT inhibited the proliferation (that is, mostly M2-M3). By contrast, the combination of FAK1 and LAT-Y171F restored to proliferation (that is, M4-M6) to that seen in the vector transfected cells (Fig. 7e, see right histogram). These observations showed that LAT Y-171 reversal of the dissociative effects of FAK1 on T-cell/DC conjugation was reflected in a restoration of T-cell proliferation.

At last, JCAM.2 T cells lacking LAT were transfected with wild-type LAT versus Y-171 and assessed for random motility on ICAM-1-Fc coated plates (Fig. 7f). Although wild-type LAT supported extensive random migration as seen by the presence of extensive tracks, Y-171 mutant arrested motility as shown by the absence of tracks (Fig. 7f, right panels). Blotting showed equal levels of expressed wild-type and mutant (Fig. 7f, left panel). These observations indicated, using another model, that the LAT Y-171 site is needed for T-cell motility, in this instance, random motility.

## Discussion

Although LFA-1 binding to ICAM-1 initiates T-cell adhesion to antigen-presenting cells, the identity of signals that limit or terminate conjugate is poorly understood. In this context, LFA-1 undergoes changes in affinity and avidity (that is, clustering), events that have been assumed to increase adhesion for conjugate formation. LFA-1 generates ‘outside-in’ co-signals, although whether these events affect TCR signalling has been unclear. Here, we show that although increased affinity initiates adhesion, LFA-1 cross-linking recruits and activates FAK1 and PYK2 to phosphorylate LAT selectively on a single Y-171 site that binds to the GRB2-SKAP1 complex and limits dwell times with DCs. Our findings made two novel points, firstly that LFA-1-FAK-1 co-signals can intersect with TCR signalling by re-structuring the LAT complex in T cells, and second, that LFA-1 is an ‘auto-regulatory on-off’ receptor that can mediate the opposing roles of adhesion and de-adhesion dependent on affinity versus clustering.

Previous studies underscored the primacy of affinity over clustering in regulation of LFA-1 adhesiveness^27^. The current model is that increased affinity cooperates with increased avidity to increase adhesion. The fact an increased LFA-1 affinity precedes clustering caused us to question whether cross-linking might generate co-signals that also terminate conjugation (that is, auto-regulatory model). In agreement, we found that the cross-linking of LFA-1 recruited and activated focal adhesion kinases FAK1/PYK2 to phosphorylate LAT Y-171 and reduce contact times between T cells and DCs. In our model, increased LFA-1 affinity to monovalent ligand ICAM1 mediates contact with DCs which is then followed by clustering and cross-linking of the receptor, which reduces or terminates adhesion. This occurs via the FAK1 activation, and its mono-phosphorylation of LAT Y-171. Since LFA-1 clusters in the pSMAC, it seems reasonable that some degree of receptor cross-linking would occur. Importantly, FAK1/PYK2 were recruited and activated by LFA-1 cross-linking, and not by affinity activation of LFA-1 with MgCl2+/EGTA, or the monovalent binding of anti-LFA-1 Fab’ or in combination. FAK1/PYK2 auto-phosphorylation or LAT Y-171 phosphorylation depended on cross-linking with a bivalent antibody. These general effects were seen in DO11.10, Jurkat and primary T cells. LFA-1 is, therefore, similar to receptors such as CD4 and CD8, which we previously showed to recruit the kinase p56^lck^ to activate signalling pathways in cells^4^,^6^,^7^. In the case of LFA-1, associated FAK1 interferes or terminates conjugate formation initiated by affinity induced LFA-1 adhesion. The pathway could also have roles during leukocyte endothelial transmigration.

Previous studies have focused on the central role played by LAT in the mediation of TCR signals^2^. Our study has identified two new kinases, FAK1 and PYK2, which can phosphorylate and interface with the LAT adaptor. Intriguingly, unlike ZAP-70 which phosphorylates multiple sites on LAT (that is, Y-132, Y-191, Y-171 and Y-226), FAK1 and PYK-2 phosphorylated only a single site on LAT at Y-171. This remarkable fidelity was seen in overexpression and in *in vitro* kinase assays, as well as with FAK1 drug inhibition following LFA-1 ligation. LFA-1-FAK1 therefore skewed the nature of LAT regulation from poly- to mono-phosphorylation at Y-171. FAK1 and PYK2 showed that same pattern consistent with the conserved nature of their kinase domains. LFA- and FAK1 in turn facilitated recruitment of a novel GRB-2-SKAP1 complex. As with GADs binding to SLP-76, we found that the SH3 domains of GRB-2 bound to SKAP1, whereas the SH2 domain of the adaptor bound to LAT. In this manner, GRB-2 brings SKAP1 to the LAT complex. FAK1 and PYK2 were also part of the complex by unexplained mechanisms.

Surprisingly, we found that LAT-GRB-2-SKAP1 complexes were distinct from LAT-GADS-SLP-76 complexes. At no point did anti-SKAP1 precipitates show the presence of either SLP-76 or GADs in response to anti-LFA-1 or anti-LFA-1/CD3, despite co-precipitating LAT, or vice versa. This new observation indicates that different receptors produce distinct LAT complexes for the regulation of function. As a possible explanation, anti-LFA-1 induced LAT-SKAP1 micro-cluster formation primarily in the pSMAC-like region enriched with LFA-1. This contrasted with anti-CD3 induced LAT-GADs-SLP-76 complexes that are seen in both the centre and periphery of cells^54^,^62^. This concentration of LAT-FAK1 might topographically allow for FAK1 mediated phosphorylation separate from ZAP-70. However, it was also noteworthy that TCR ligation cooperated strongly with anti-LFA-1 with increased LAT-SKAP1 proximity as seen by *in situ* proximity analysis and biochemically by the presence of co-precipitated SKAP1. This basis for this cooperation is not clear, but might be due to the well-known ability of the TCR to promote LFA-1 clustering and to independently induce FAK1 and PYK2 auto-phosphorylation that could synergize with anti-LFA-1.

Functionally, we found that FAK1 reduced T-cell contact times with DCs as observed by siRNA downregulation of FAK1 (that is, which increased dwell times), or by FAK1 transfection (that is, which reduced contact times). Increased dwell times in turn correlated with reduced motility, and conversely, reduced contact with increased motility. Intriguingly, co-expression of the LAT-Y171F mutant reversed the ability of FAK1-GFP to shorten conjugation times. In fact, in the absence of co-transfected FAK1, it even increased mean contact times relative to the vector transfected cells, presumably due to an ability to complete with endogenous wild-type LAT, resulting in increased contact times and enhanced proliferation at wild-type levels. These findings are therefore consistent with a model where LAT Y-171 residue negatively regulates T-cell conjugation formation and activation when phosphorylated by FAK1. Whether PYK2 operates the same way is uncertain. Reconstitution studies showed that PYK2 failed to replace FAK1 to enhance migration^63^.

Further downstream, the full range of signalling events responsible for the reduced conjugation of T cells remains to be determined. We previously showed that SKAP1 regulates LFA-1 mediated adhesion, conjugation and motility^32^,^34^,^37^,^39^. In this context, SKAP1 expression is needed for TCR induced Rap1-RapL complex formation, whereas RapL mutations that abrogate SKAP1 binding reverse its ability to reduce T-cell motility (that is, stop signal) in LNs^37^. We also previously showed that the expression of the SKAP1 binding partner, ADAP promoted the motility of T cells on ICAM1 coated plates^19^. At the same time, FAK1 promotes the turnover of cell contacts in non-lymphoid cells lacking ADAP and SKAP1 (refs 48, 64, 65). FAK1-deficient cells are less motile with larger focal adhesion plaques^48^. Downstream regulators may therefore also include other FAK1 targets such as a focal adhesion associated protein, paxillin or RhoA^66^. LAT-GRB-2 also binds to THEMIS, a mediator of cell survival^67^.

LAT has multiple potential sites for GADs and GRB-2 binding as well-established by the Samelson lab^2^. GRB-2 and GADs use their SH2 domains to bind LAT and their SH3 domains to bind to their respective binding partners. We found that the N-terminal SH3-1 domain was most effective in binding to the SK region of SKAP1 (between the SH3 and PH domains). The GADs SH3 domain binding is unusual in binding to an atypical motif on SLP-76 (ref. 17), that is not present in SKAP-1.

The conditions that determine the specificity of SH2 binding to LAT are not fully understood. GRB-2 binding requires an N in the plus 2 position relative to the Y that are found in sites, Y-110 (YENE), Y171 (YVNV), Y191 (YVNV) and Y-226 (YENL)^68^. These motifs correspond to a class of hybrid ligand motifs that are permissive and less selective for GRB2 binding as seen with the CD28 pYMNM motif that binds to both GRB-2 and PI 3-kinase^69^,^70^. The putative sites are not equally bound or accessible to GRB-2 (or GADs)^59^. With LFA-1, we found that GRB-2-SKAP1 bound only to the Y-171 site, the site phosphorylated by FAK1/PYK2. Further, FAK1 and PYK-2 were also part of the complex as seen in co-precipitation experiments. It is possible that the presence of these other components promotes the preferential binding of GRB-2 for Y-171 motif.

Overall, our findings fit a new model (that is, auto-regulatory) where LFA-1 mediates both adhesion and de-adhesion. LFA-1 co-signals intersect with TCR signals at the level of the LAT complex. In our model, adhesion is mediated by increased affinity, whereas the subsequent clustering and cross-linking of LFA-1 leads to FAK1/PYK2 activation and the phosphorylation of LAT to limit or terminate T-cell/DC conjugation in adaptive immune responses. Enhanced TCR ligation at the nascent IS would activate ZAP-70 to induce classic activating LAT- PLCγ1/SLP-76 complexes. ITK, RLK and ACK (Cdc42-associated kinase 1) phosphorylate SLP-76 (refs 71, 72, 73), whereas PLCγ1 and GADs-SLP-76 mobilize calcium and activate the ERK pathway^9^,^11^. However, with the maturation of the IS and subsequent clustering, the cross-linking of LFA-1 (either alone or in conjunction with the TCR) would become increasing dominant with increased numbers of LAT-GRB-2 SKAP1 complexes at the expense of activating LAT-GADs-SLP-76- complexes. Further, by activating FAK1, LFA-1 would induce a pathway that leads to reduced conjugation and the dissociation of T cells from DCs. Our findings suggest the possible use of FAK1 inhibitors to increase T-cell conjugate formation in vaccine development and anti-tumour immunotherapy.

## Methods

### Cell culture

Jurkat cells (American Type Culture Collection (ATCC)) were grown in RPMI meeium 1640 with 10% fetal calf serum (FCS), 2 mM L-glutamine, penicillin and streptomycin. J14 cells (SLP-76-deficient Jurkat cells) were kindly provided by Dr A. Weiss (UCSF). DC27.10 cells were obtained from Dr Oreste Acuto (University of Oxford), whereas isolated mouse splenocytes were cultured after removal of red blood cells with hypotonic buffer (0.15 M NH_4_Cl, 1 mM NaHCO_3_, 0.1 mM EDTA, PH 7.25) in RPMI containing 10% FCS, 5% glutamine, 5% penicillin/streptomycin and 2-mercaptoethanol at 5 × 10^−5^ M. To generate T-cell blasts, isolated splenocytes were cultured with Con A (2.5 μg ml^−1^) for 2 days, and then cultured in growth medium with interleukin-2 (IL-2) (20 ng ml^−1^) for 2–3 days. For experiments, T cells were washed and rested in growth medium for 2 days in the absence of growth factor. CD4^+^ T cells were purified using Dynabeads mouse CD4 (11415D–Thermo Fisher Scientific) according to the manufacturer's instructions (Invitrogen). All experiments abided by the ethical review standards (Research Ethics Committee and the Animal Welfare and Ethical Review Body) of Cambridge University.

Bone marrow-derived dendritic cells (BMDC) were cultured at 1 × 10^6^ cells per ml^−1^ in RPMI medium supplemented with 10% FCS, 2 mM glutamine, 100 IU ml^−1^ penicillin, 100 mg ml^−1^ streptomycin and 50 M 2-mercaptoethanol, 20 ng ml^−1^ recombinant murine GM-CSF and 10 ng ml^−1^ IL-4. On day 7 of culture, BMDCs were induced to mature by adding 1 mg ml^−1^ LPS to the medium. After an overnight incubation, non-adherent cells and loosely adherent proliferating BMDC aggregates were collected, washed and replated for 1 h at 37 °C to remove contaminating macrophages.

Human T cells were prepared from buffy coats isolated by leukophoresis (Addenbrookes Hospital Cambridge with donor consent). Mononuclear cells were isolated by Ficoll density gradient centrifugation. After being washed, cells were stimulated for 24 h at 37 °C with 5 μg ml^−1^ of phytohemagglutinin. After two washes, cells were maintained for 5–6 days in exponential growth phase in RPMI medium plus 10% FCS supplemented with 20 ng ml^−1^ of recombinant IL-2, followed by washing and IL-2 starvation for 24 h. Cells were re-suspended at 10^8^ cells per ml in 250 μl of complete medium containing 100 μg of SLP 76 EYFP expression construct.

Transfections were performed in 4 mm gap-cuvettes with the use of a BTX ECM 830 electroporator with a single pulse of 385 V and 6 ms. Cells were immediately transferred to pre-warmed complete medium and allowed to recover for 24 h prior to analysis. siRNAs specific for FAK1 (not PYK2) and control scrambled siRNAs were synthesized by Cell Signaling Technology. FAK siRNA purchased from Santa Cruz Biotechnology, Cat No: sc-35353. No effect of FAK-1 siRNA was noted on PYK2 expression.

### Antibodies

Anti-human CD3 (OKT3) (#IMG 6240E) was obtained from Imgenex, anti-mouse CD3 (145-2C11) ) (# 553057) was obtained from BD Pharmingen. CXCL12/SDF-1α (#460-SD/CF) was purchased from R&D. Anti-LAT (#06-807), anti-Tyr402-PYK2 (#07-892), anti-FAK (#05-537), anti-p397-FAK (05-1140) and SKAP1(#07-651) were purchased from Millipore. Anti-pY-191-LAT (#3584), anti-p171-LAT (#3581), Anti-pY226-LAT (#07-295), anti-ADAP (#07-546), anti-GRB-2 (#3972), anti-Myc (clone 9B11) (#2276), anti- PYK2 (#06-559) and anti-GADS (#06-983) were purchased from Cell Signaling. Anti-pY132-LAT (#44-224) was from Biosource. Anti-GFP (clone B-2) (sc-9996) and anti-GST (clone B-14) (#sc-138) were from Santa Cruz. Anti-Flag (#F4799) was from Sigma. Anti LFA-1(clone M18/2) (#557437) was purchased from BD Pharmingen and anti-ICAM-1(#796-IC) were purchased from R &D. Alexa-488 conjugated goat anti-rabbit IgG and Alexa-568 conjugated goat anti-mouse IgG1 were purchased from Invitrogen. Poly-L-lysine, pertussis toxin and B subunit were bought from Sigma, chambered coverslips from Nalge Nunc. Anti-SKAP1 (clone 35) (#611236) was from BD Transduction Laboratories, anti-V5 (#R960-25) (Invitrogen), anti-RapL (#18-001-30078) (GenWay Biotech, Inc.), and anti-β-actin (clone AC-74) (#A2228) (Sigma) were purchased as assigned. (All antibodies were used with a dilution of 1:1,000). Anti LFA-1 and anti-ICAM-1 were purchased from R &D. Alexa-488 conjugated goat anti-rabbit IgG and Alexa-568 conjugated goat anti-mouse IgG1 were purchased from Invitrogen. Poly-L-lysine, pertussis toxin and B subunit were bought from Sigma, chambered coverslips from Nalge Nunc.

### Constructs and transfection

Constructs of SKAP1 and GRB-2 were inserted into a pGEX5x-3 (GE Healthcare), into a 3xFlag-tagged and EGFP-tagged pcDNA3.1-Hygro (Invitrogen) vector. LAT and various mutants were cloned into a myc and 3xFlag-tagged pcDNA3.1 vector. PYK2 and FAK were cloned in a 3xFlag-tagged vector. SKAP1 and PLCgamma1 were cloned in pSRalpha expression vector in-frame with HA-tag. SLP-76 cDNA was sub-cloned in the pEYFP-N1 vector (Clontech). Mutants were generated by site-directed mutagenesis using the Quick Change protocol and Pfu Ultra II Fusion HS DNA Polymerase (Stratagene). All constructs were confirmed by sequencing. Transfections were performed in 4mm gap-cuvettes with the use of a BTX ECM 830 electroporator with a single pulse of 385 V and 6 ms. Cells were immediately transferred to pre-warmed complete medium and allowed to recover for 24 h prior to analysis. FAK and PYK inhibitor PF 431396 and PF 573228 were purchased from Tocris.

### *In situ* PLA

*In situ* PLA was achieved using DuolinkTM *in situ* PLA reagents. Cells on slides were blocked with Duolink Blocking stock followed by the application of two PLA probes in 1x Antibody Diluent. Wash the slides in a wash buffer (1 × TBS-T) for 5 min, twice and carry out a hybridization using Duolink Hybridization stock 1:5 in high-purity water and mix followed by incubation for 15 min at +37 °C. Duolink Ligation with ligase and incubation of the slides in a pre-heated humidity chamber for 15 min at +37 °C. Amplification was then achieved using Duolink Amplification stock and polymerase with incubation of the slides in a pre-heated humidity chamber for 90 min at +37 °C. The quantification was based on the counts of five different fields in each experiment (*n*=4).

### Immunoblotting

Precipitation was conducted by incubation of the lysate with the antibody for 1 h at 4 °C, followed by incubation with 30 μl of protein G-Sepharose beads (10% w/v) for 1 h at 4 °C as described^8^,^37^,^38^. Immunoprecipitates were washed three times with ice-cold lysis buffer and subjected to SDS-PAGE. For blotting, precipitates were separated by SDS-PAGE and transferred onto nitrocellulose filters (Schleicher and Schuell). Bound antibody was revealed with horseradish peroxidase-conjugated rabbit anti-mouse antibody using enhanced chemiluminescence (Amersham Biosciences). For purification of membrane fractions, Jurkat, DC27.10 or primary T cells were sheared in hypotonic buffer and the nuclei removed by low-speed centrifugation (1,500 r.p.m., 10 min), and the supernatant was recentrifuged at high speed (25,000 r.p.m.) for 1 h. The cytosolic fraction comprised the supernatant, whereas membranes remained in the pellet. The full length versions of the immunoblots in the paper is shown in Supplementary Fig. 2.

### GST pull-down assay

The expression of recombinant GST-proteins was induced in *Escherichia coli* BL21 cells at 37 C for 2 h by the addition of 1 mM IPTG. GST-fused proteins were purified with the Cell Lytic B protocol (Sigma #B7435). Cell lysates were incubated with GST fusion proteins for 3 h followed by analysis with SDS-PAGE and western blotting.

### Integrin adhesion assay

Jurkat cells (1–2 × 10^5^ cells per well) were incubated with 5 mM MgCl^2+^ and 1 mM EGTA for 15 min, were washed and assessed for adhesion to immobilized ICAM-1-Fc-coated plates by incubating cells on plates for 30 min at 37 °C. Flat-bottomed 96-well plates had been coated previously with 4 μg ml^−1^ murine ICAM-1 human Fc in PBS overnight at 4 °C, washed with RPMI 1640 medium, and blocked with 2.5% BSA in PBS for 1 h at 37 °C. Plates with adhered cells were then gently washed twice with RPMI 1640 medium followed by the counting of cells and photography of the plates^74^.

To measure the effect of soluble antibody or ICAM1-Fc binding on cells, Jurkat cells were incubated with MgCl_2_/EDTA, soluble anti-LFA-1 Fab’ (1 μg ml^−1^), soluble Fab’ plus MgCl_2_/EDTA or soluble anti-LFA-1 (1 μg ml^−1^), for various times followed by blotting with anti-pY171- LAT, anti-LAT, anti-pY-397-FAK, anti-FAK or anti-pY-402-PYK2 and anti-PYK2. Similarly, cells were incubated with soluble mono-valent ICAM1-Fc alone (2 μg ml^−1^), or in combination with ICAM1-Fc MgCl^2+^/EGTA over 5–20 min followed by blotting with antibodies to Y-171 LAT or pY401-PYK2. The assay was performed in the absence of ICAM1 on plates.

### Live cell imaging

Jurkat T cells were transfected with LAT-cherry and SKAP1-GFP, and were imaged on anti-CD3 or anti-CD3-ICAM1-Fc coated cover slips. All imaging assays were performed in Poly-L-lysine-treated chambered glass culture slides (Lab-tek) as described^56^,^57^. Plates were coated with 5 μg ml^−1^ anti-CD3 (OKT3) and 2 μg ml^−1^ ICAM1-Fc overnight at 4 °C. Cells were imaged at the interface using a Zeiss LSM 510 confocal microscope using excitation wavelengths of 514 nm for EYFP and 594 nm for mcherry and a × 63 oil immersion objective (NA=1.2). Images were collected at 10 s intervals. Single Z sections were captured over time to improve the rate of image acquisition. All images were processed by Volocity software (Improvision). Standard deviations and standard errors were calculated with the use of Microsoft Excel and GraphPad Prism. Differences between means were tested using unpaired Student’s *t-* test.

### Statistical analysis

Pearson’s correlation coefficients (Rr) were calculated by intensity correlation analysis with ImageJ. Column statistics were performed with GraphPad software (Prism). Paired and unpaired *t-*tests were performed to analyse the data where appropriate. In certain instances, one-way analysis of variance between groups followed by a series of *t-*tests. Mean values are shown and error bars represent the s.e.m. In the statistical analysis, *P* values >0.05 are indicated as nonsignificant (ns), *P* values between 0.01 and 0.001 are indicated by double asterisks (**), and *P* values smaller than 0.001 are indicated by triple asterisks (***).

### Data availability

Data supporting the findings in this study are available within this article and from the corresponding author upon reasonable request.

## Additional information

**How to cite this article:** Raab, M. *et al*. LFA-1 activates focal adhesion kinases FAK1/PYK2 to generate LAT-GRB2-SKAP1 complexes that terminate T-cell conjugate formation. *Nat. Commun.* **8,** 16001 doi: 10.1038/ncomms16001 (2017).

**Publisher’s note:** Springer Nature remains neutral with regard to jurisdictional claims in published maps and institutional affiliations.

## Supplementary Material

Supplementary Information
