# Supplementary material for: LFA-1 activates focal adhesion kinases FAK1/PYK2 to generate LAT-GRB2-SKAP1 complexes that terminate T-cell conjugate formation
Source: Nat Commun. 2017 Jul 12;8:16001. doi: 10.1038/ncomms16001 (PMC5510181; doi:10.1038/ncomms16001)
Supplement: Supplementary Information [file ncomms16001-s1.pdf]

Title of file for HTML: Supplementary Information
Description: Supplementary Figures

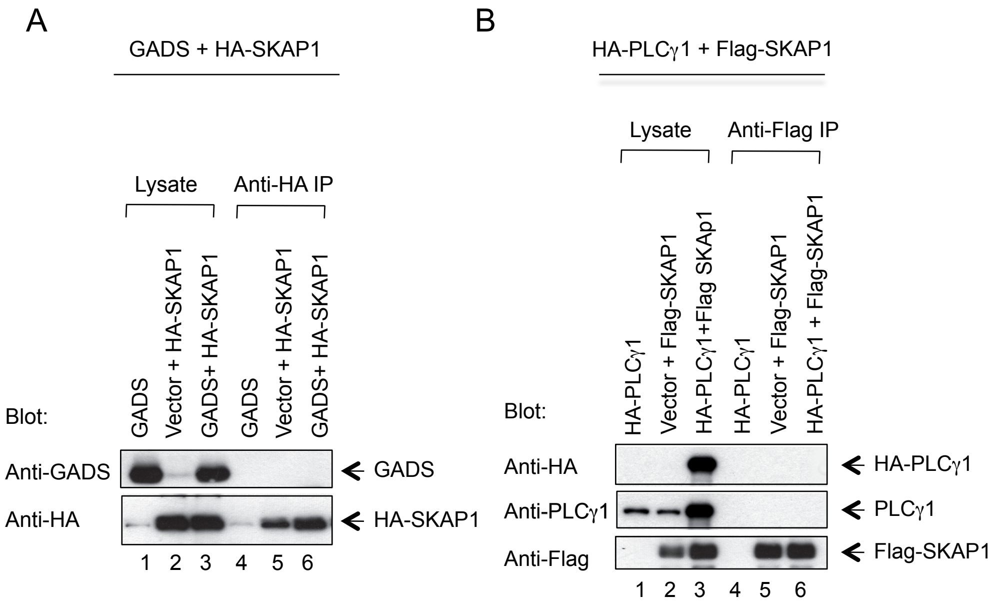

Supplementary Figure 1.
(A) GADs does not bind to SKAP1. 293T cells were transfected as above with GADs, HA-SKAP1 or both, followed by anti-HA precipitation and blotting with anti-GADs or anti-HA. Lanes 1-3: cell lysates; lanes 4-6: anti-HA precipitations. (**B) PLCγ1 does not bind to SKAP1.** 293T cells were transfected with HA-PLCγ1, FLAG-SKAP1 or both, followed by anti-Flag precipitation and blotting with anti-HA, anti-PLCγ1 or anti-Flag. Lanes 1-3: cell lysates; lanes 4-6: anti-Flag precipitations.

Figure 1C

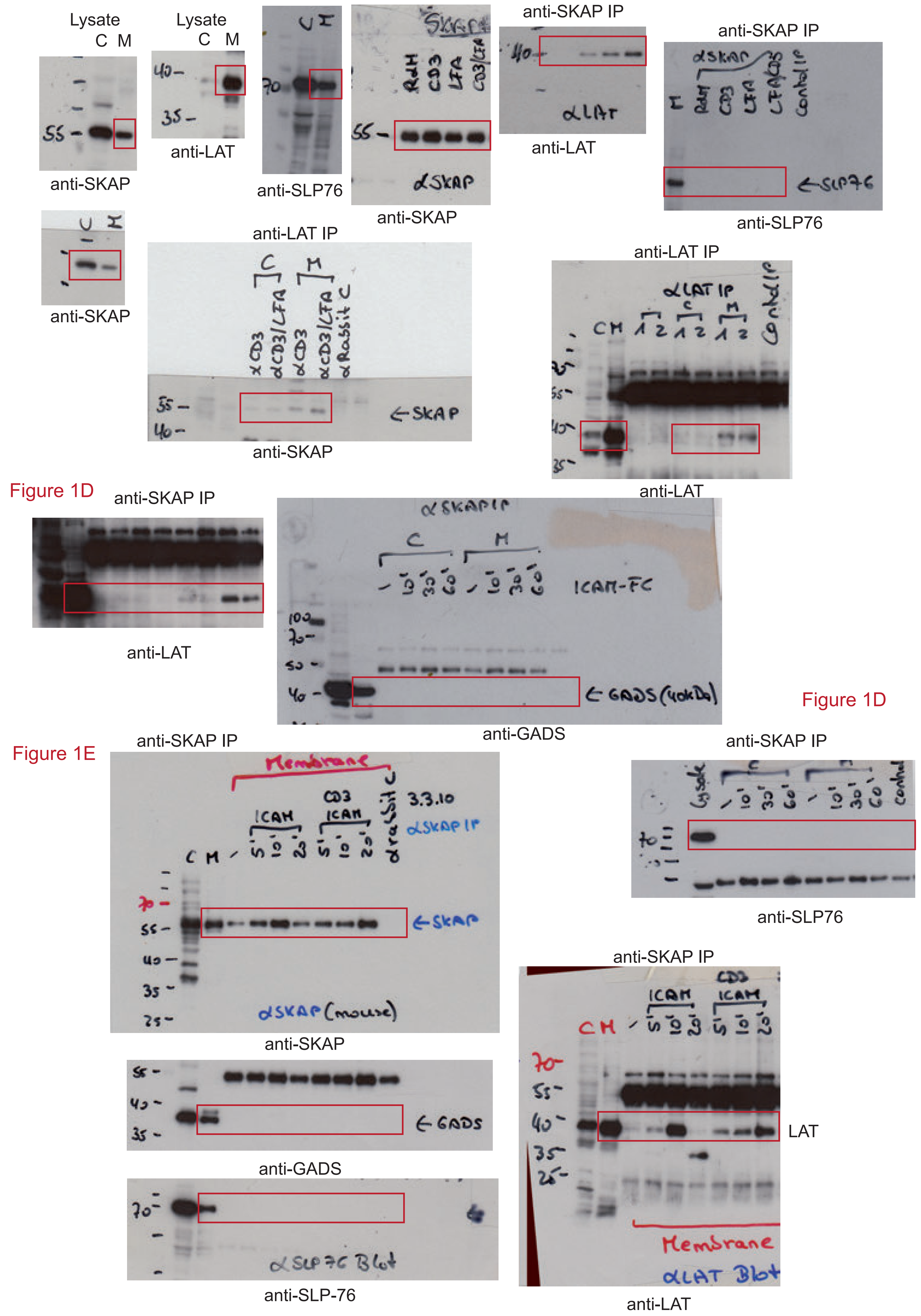
Lysate
C M
anti-SKAP
Lysate
C M
anti-LAT
anti-SLP76
anti-SKAP IP
anti-SKAP
anti-SKAP IP
anti-LAT
anti-SKAP IP
anti-SLP76
anti-SKAP
anti-LAT IP
anti-SKAP
anti-LAT IP
anti-LAT
Figure 1D
anti-SKAP IP
anti-LAT
anti-GADS
Figure 1D
anti-SKAP IP
anti-SLP76
Figure 1E
anti-SKAP IP
anti-SKAP
anti-GADS
anti-SLP-76
anti-SKAP IP
LAT
anti-LAT

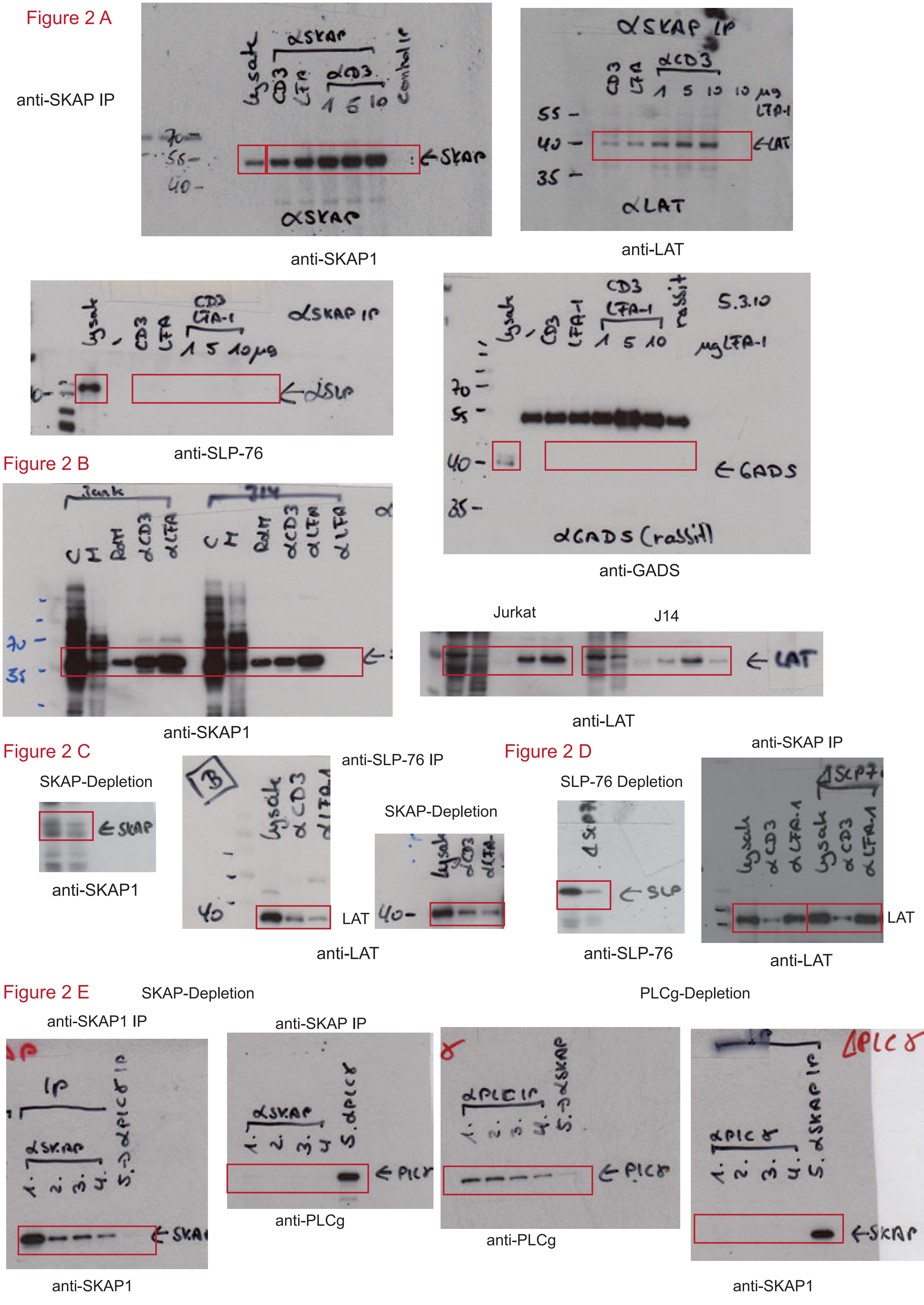
Figure 2 A
anti-SKAP IP
anti-SKAP1
anti-LAT
anti-SLP-76
anti-GADS
Figure 2 B
Jurkat
J14
anti-SKAP1
anti-LAT
Figure 2 C
SKAP-Depletion
anti-SKAP1
anti-SLP-76 IP
SKAP-Depletion
LAT
anti-LAT
Figure 2 D
SLP-76 Depletion
anti-SKAP IP
anti-SLP-76
LAT
anti-LAT
Figure 2 E
SKAP-Depletion
PLCg-Depletion
anti-SKAP1 IP
anti-SKAP IP
anti-PLCg
anti-PLCg
anti-SKAP1
anti-SKAP1

Figure 2 F

anti-SKAP IP

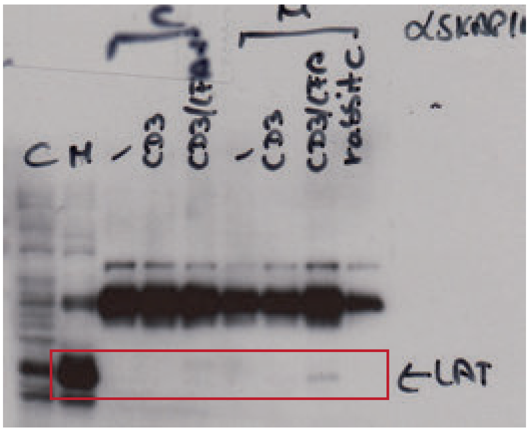

anti-LAT

anti-SKAP IP

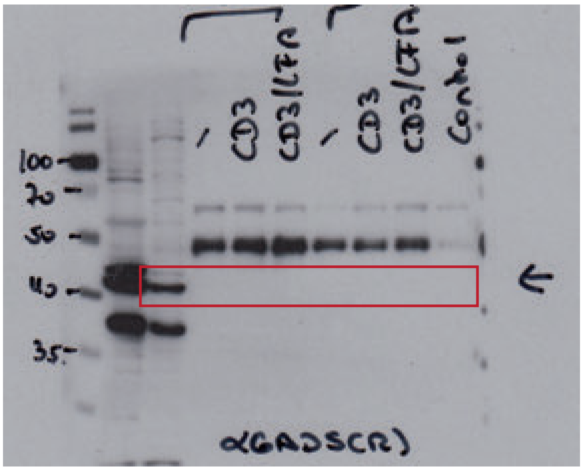

anti-GADS

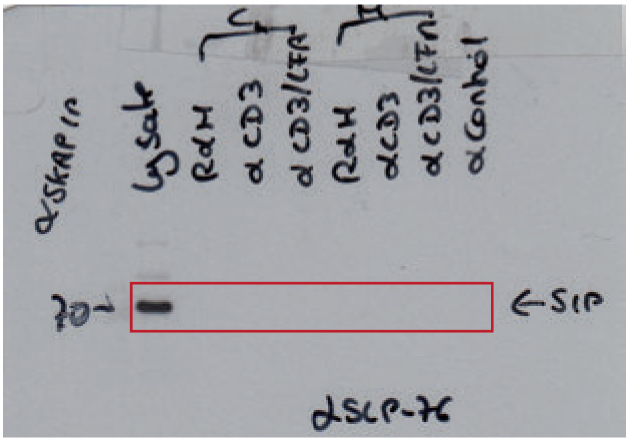

anti-SLP-76

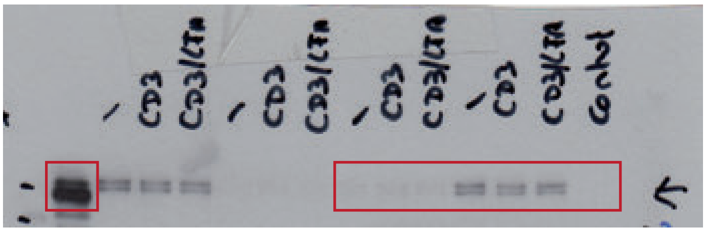

anti-ADAP

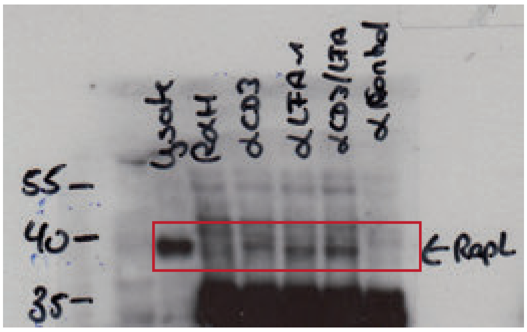

Figure 3 A

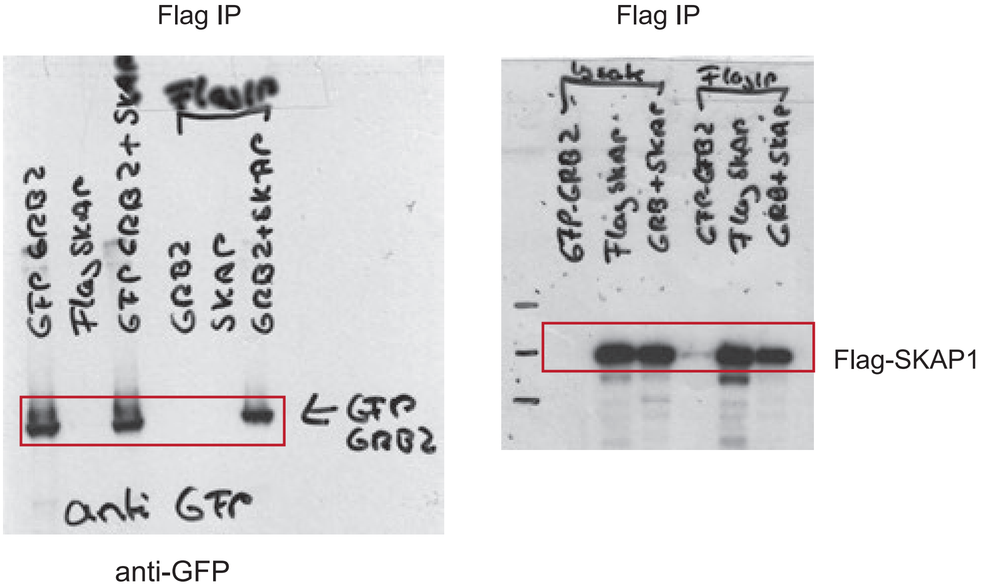

Figure 3 B

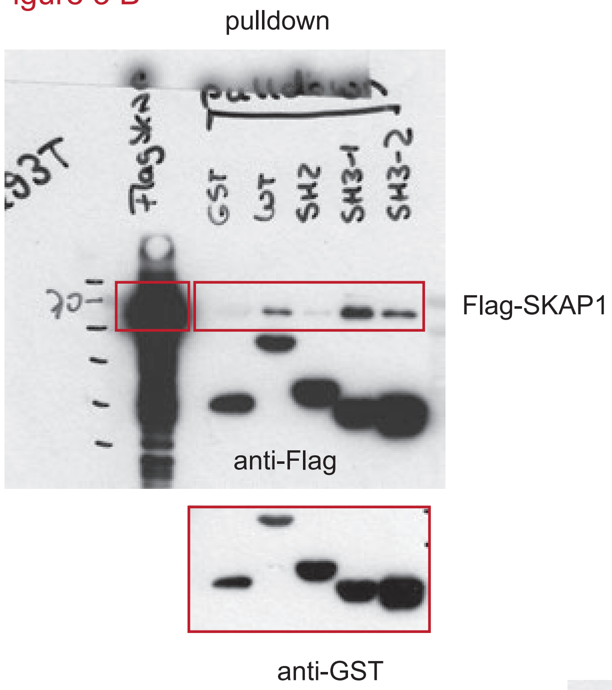

Figure 3 C

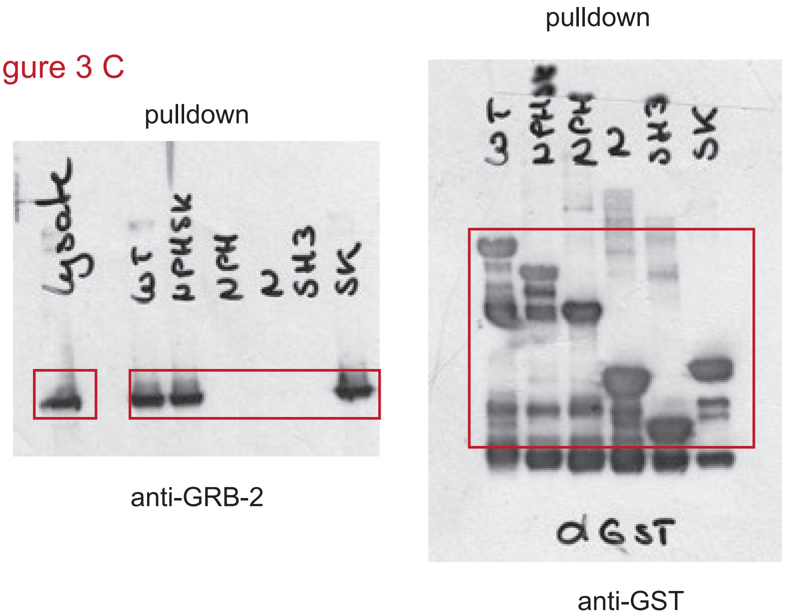

Figure 3 D

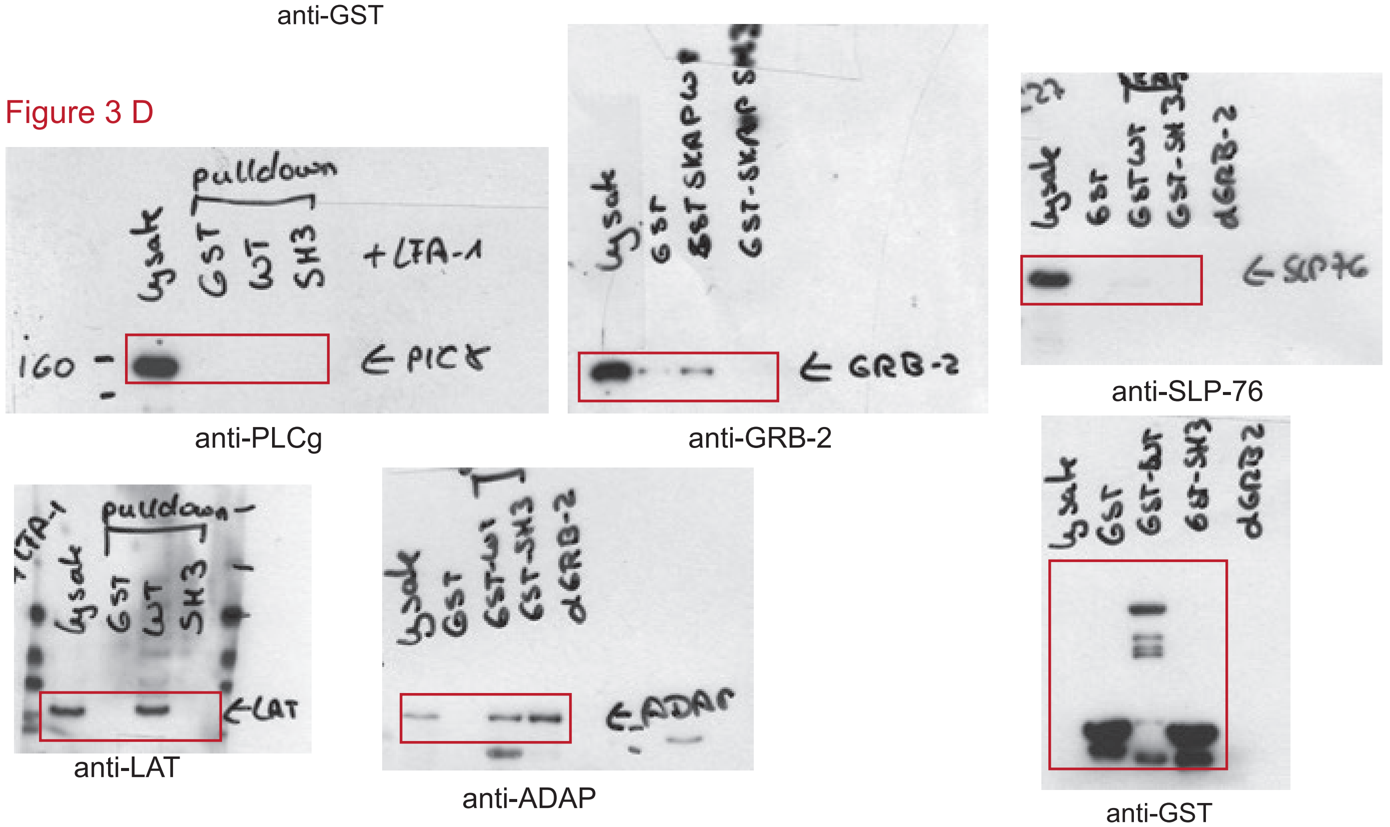

Figure 4 A

Time kinetic (membrane)

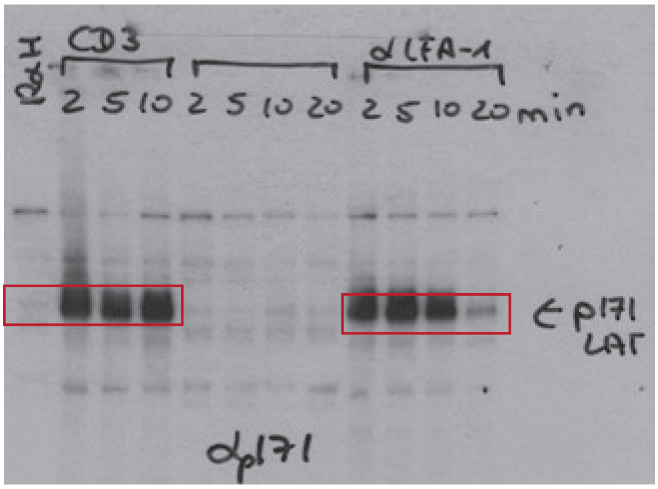

anti-p171-LAT

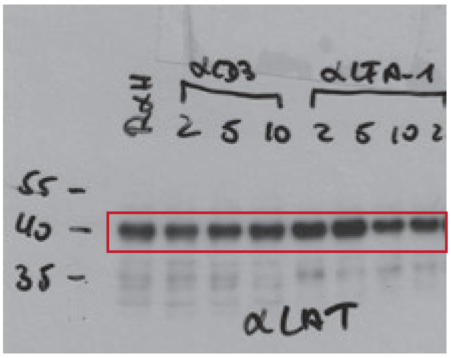

anti-LAT

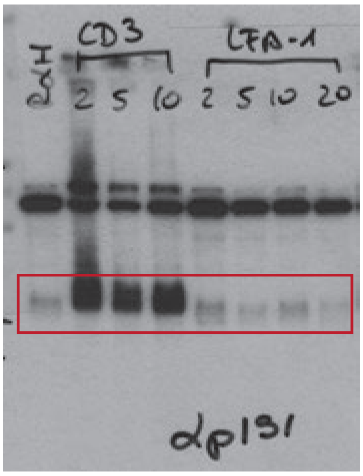

anti-p191-LAT

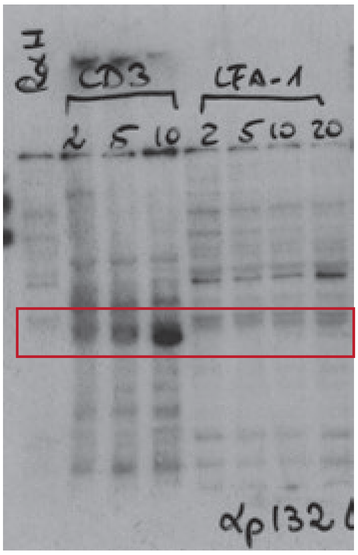

anti-p132-LAT

Figure 4 B

anti-LFA-1 IP

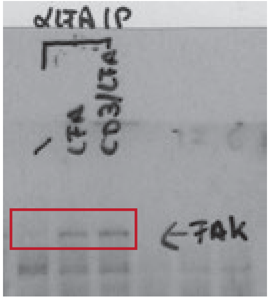

anti-FAK1

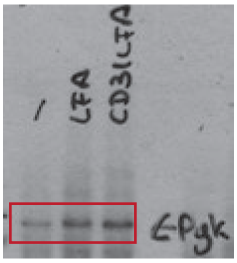

anti-Pyk2

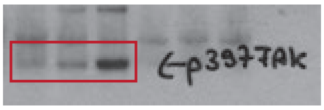

anti-p397-FAK1

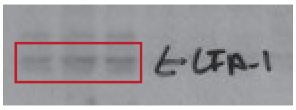

anti-LFA-1

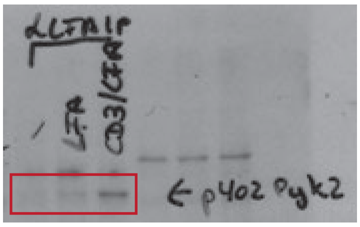

anti-p402-Pyk2

Figure 4 C

Kinase Assay

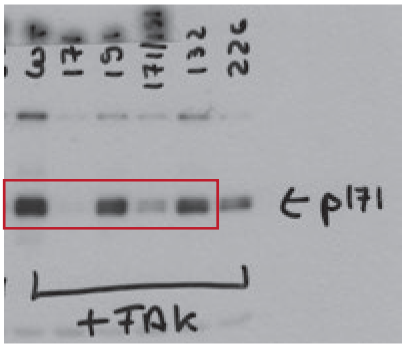

anti-p171-LAT

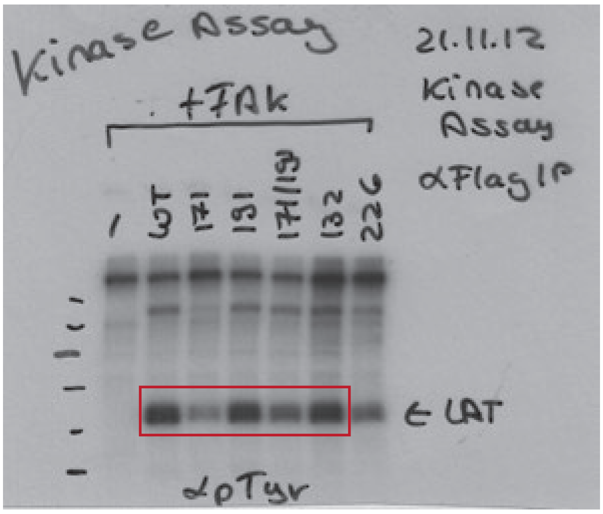

anti-pTyr

Lysate

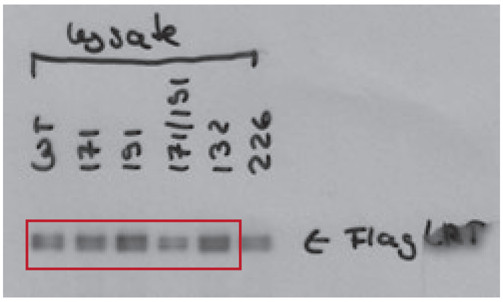

anti-Flag

anti-Flag IP

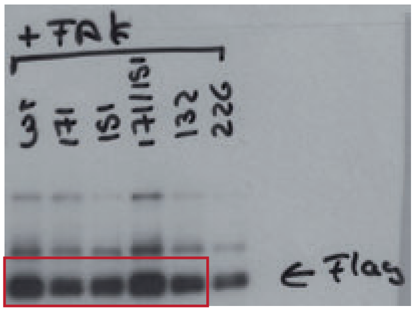

anti-Flag

Figure 4 D

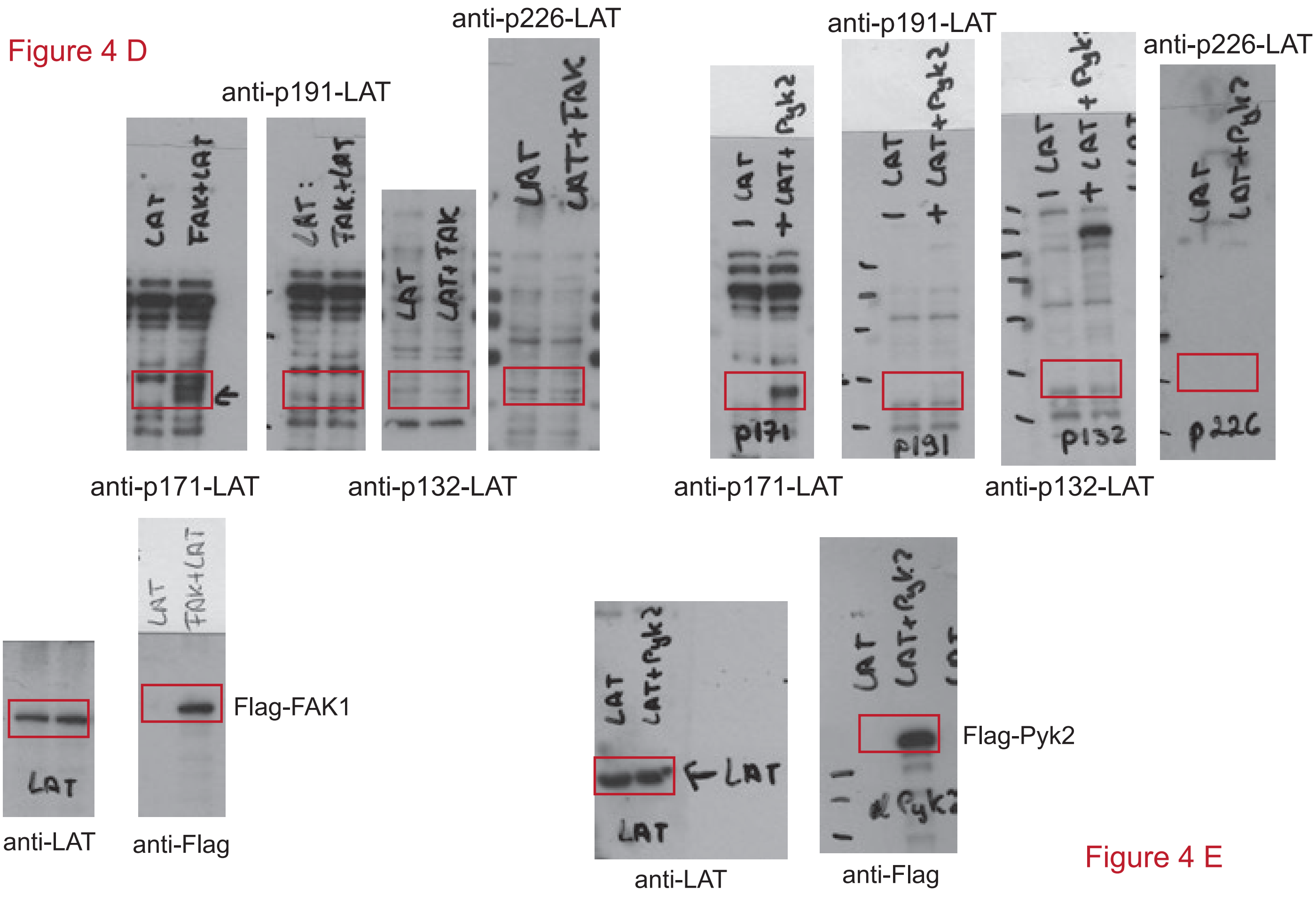

Figure 4 F

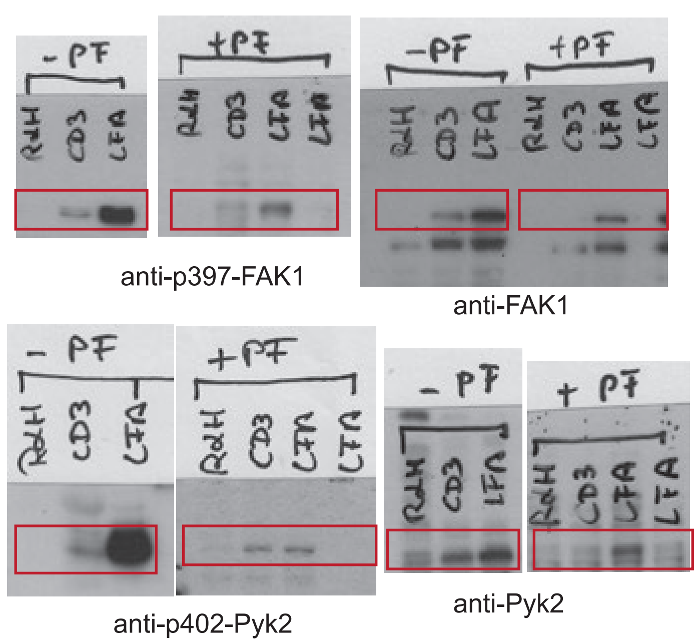

Figure 4 E

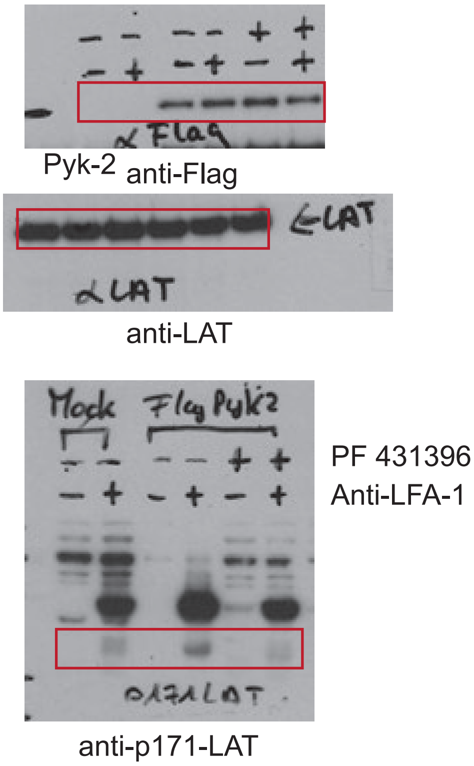

Figure 4 H

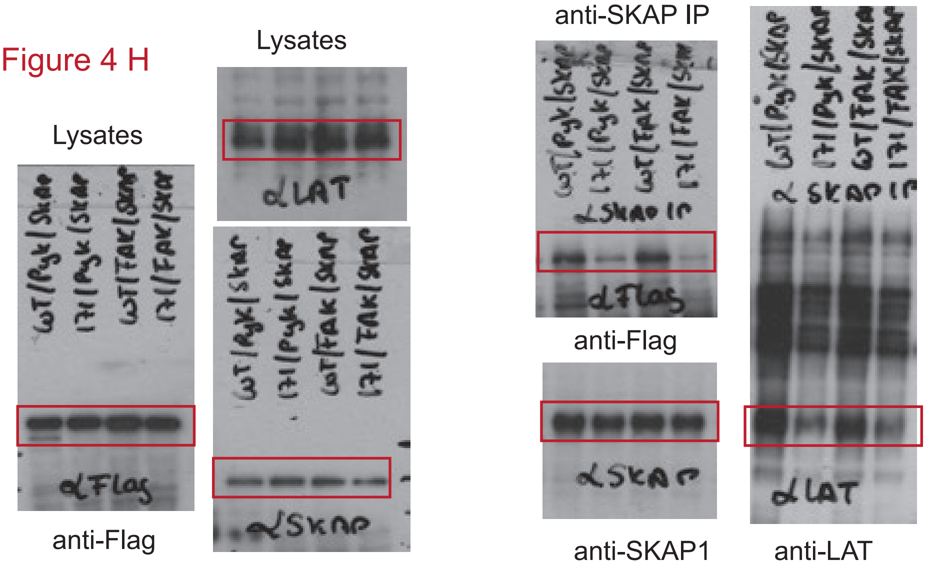

Figure 4 G

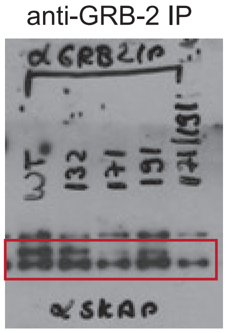

Figure 5 B

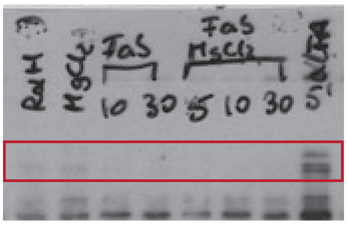

anti-pPyk2

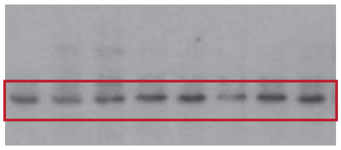

anti-FAK1

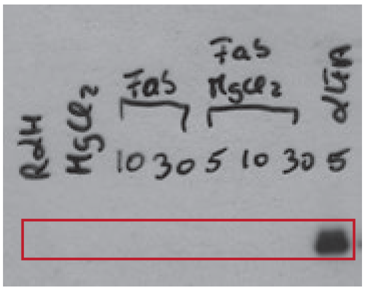

anti-pLAT

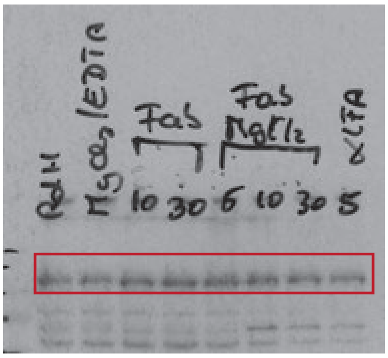

anti-Pyk2

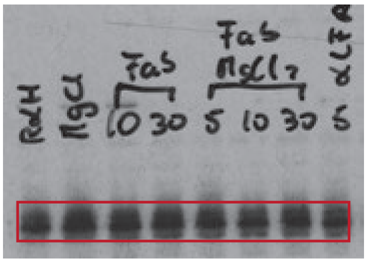

anti-LAT

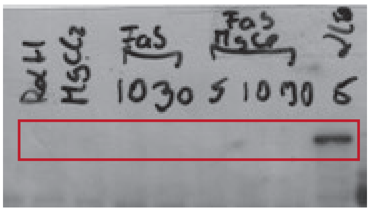

anti-pFAK1

Figure 5 C

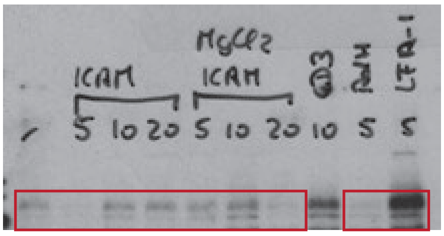

anti-pPyk2

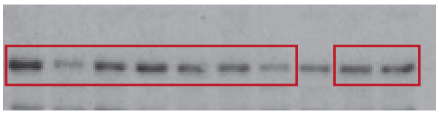

anti-pPyk2

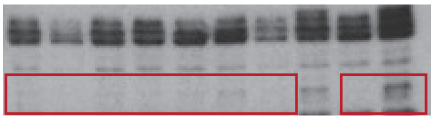

anti-pLAT

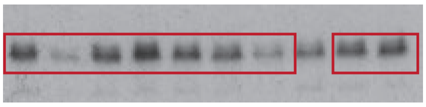

anti-LAT

Figure 6 A

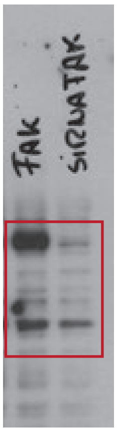

anti-FAK1/β-Actin

Figure 7 F

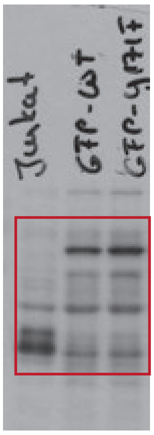

anti-LAT

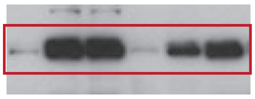

anti-HA

Suppl.Figure 1A

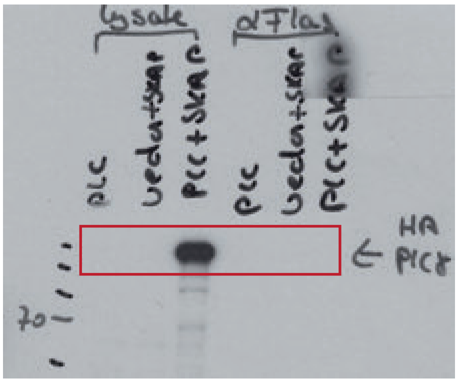

anti-HA

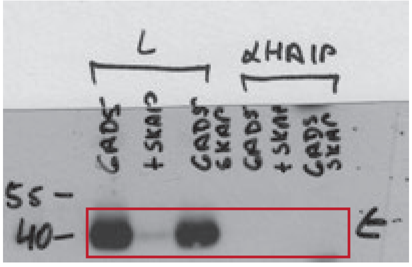

anti-GADS

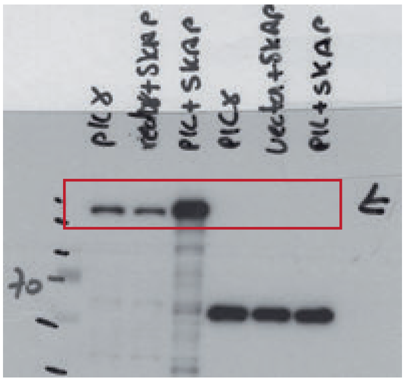

anti-PLCg1

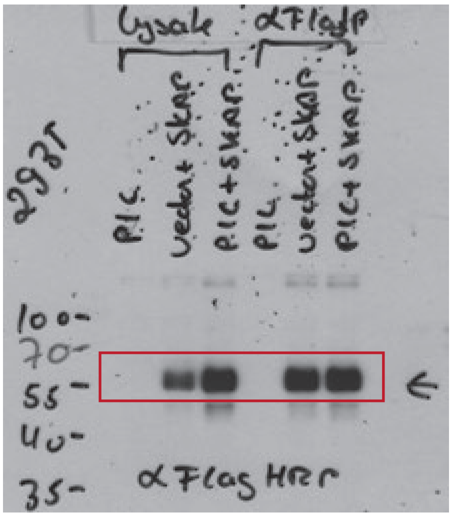

Suppl.Figure 1B

Supplementary Figure 2. Uncropped western blot images.
